# Effectiveness of mesenchymal stem cell-conditioned medium in bone regeneration in animal and human models: a systematic review and meta-analysis

**DOI:** 10.1186/s13619-020-00047-3

**Published:** 2020-06-02

**Authors:** Maria Paula Benavides-Castellanos, Nathaly Garzón-Orjuela, Itali Linero

**Affiliations:** 1 Research Group of Oral and Maxillofacial Surgery, Faculty of Dentistry, Universidad Nacional de Colombia, Bogotá, Colombia; 2 Research Group on Equity in Health, Faculty of Medicine, Universidad Nacional de Colombia, Bogotá, Colombia; 3 Research Group of Oral and Maxillofacial Surgery, Faculty of Dentistry, Research Group of Stem Cell Biology, Faculty of Medicine, Universidad Nacional de Colombia, Bogotá, Colombia; 4 Faculty of Dentistry, Universidad Nacional de Colombia, Ciudad Universitaria, Edificio 210, Bogotá, Colombia

**Keywords:** Bone regeneration, Conditioned medium, Mesenchymal stem cell, Secretome, Regenerative medicine

## Abstract

**Background:**

Given the limitations of current therapies for the reconstruction of bone defects, regenerative medicine has arisen as a new therapeutic strategy along with mesenchymal stem cells (MSCs), which, because of their osteogenic potential and immunomodulatory properties, have emerged as a promising alternative for the treatment of bone injuries. In vivo studies have demonstrated that MSCs have a positive effect on regeneration due to their secretion of cytokines and growth factors that, when collected in conditioned medium (MSC-CM) and applied to an injured tissue, can modulate and promote the formation of new tissue.

**Objective:**

To evaluate the effectiveness of application of conditioned medium derived from mesenchymal stem cells in bone regeneration in animal and human models.

**Methods:**

We conducted a systematic review with a comprehensive search through February of 2018 using several electronic databases (MEDLINE, EMBASE, SCOPUS, CENTRAL (Ovid), and LILACS), and we also used the “snowballing technique”. Articles that met the inclusion criteria were selected through abstract review and subsequent assessment of the full text. We assessed the risk of bias with the SYRCLE and Cochrane tools, and three meta-analyses were performed.

**Results:**

We included 21 articles, 19 of which used animal models and 2 of which used human models. In animal models, the application of MSC-CM significantly increased the regeneration of bone defects in comparison with control groups. Human studies reported early mineralization in regenerated bones, and no bone resorption, inflammation, nor local or systemic alterations were observed in any case. The meta-analysis showed an overall favorable effect of the application of MSC-CM.

**Conclusions:**

The application of MSC-CM to bone defects has a positive and favorable effect on the repair and regeneration of bone tissue, particularly in animal models. It is necessary to perform additional studies to support the application of MSC-CM in clinical practice.

## Background

Reconstruction of bone defects generated by fractures, tumors, infections or congenital diseases is a real challenge in oral and maxillofacial surgery and orthopedics. Although bones have an ability to repair and regenerate, in bone lesions of large size, the process of healing fails, and such injuries do not repair themselves spontaneously (Oryan et al., 2013). Current therapies have focused on the placement of grafts and bone substitutes, which are widely used but also have some limitations and disadvantages in reconstruction of bone defects that exceed the critical size (Oryan et al., 2013; D, 2010; Shrivats et al., 2014). This has stimulated the search for new therapeutic alternatives to produce adequate regeneration and rehabilitation of these defects (Padial et al., 2015; Bertolai et al., 2015). This may give rise to regenerative medicine, which seeks to repair or replace damaged cells and tissues of an organ to restore its normal functioning. Regenerative medicine uses tools from tissue engineering, gene therapy and cellular therapy, the latter of which is mainly represented by the use of mesenchymal stem cells (MSC) (de Santana et al., 2015; Berthiaume et al., 2011; Tatullo et al., 2015). MSCs are a type of adult stem cells, which are multipotent and thus can self-regenerate, proliferate and differentiate into multiple cell lineages (Saeed et al., 2016; Wen et al., 2013; Monaco et al., 2011). There have been multiple reports in the literature revealing the therapeutic effects of the application of MSC for bone regeneration in animal and human models (Cancedda et al., 2007; Ramamoorthi et al., 2015; Wang et al., 2012a). Currently, it has been suggested that their main mechanism of action in tissue regeneration and repair through the release of growth factors, cytokines and extracellular matrix molecules, which have a paracrine effect on host cells, modulating endogenous cell migration, angiogenesis, and cell differentiation, and inducing the repair and regeneration of injured tissues (Liang et al., 2014; Ivanova et al., 2016; Chaparro & Linero, 2016; Linero & Chaparro, 2014). Secreted factors are referred to as a secretome and can be found in the medium where the mesenchymal stem cells are cultivated, known as a conditioned medium (MSC-CM). It has been shown that MSC-CM exerts a beneficial effect on regeneration of bone and tissue, as the secretome participates in stimulation of multiple cellular functions (Ivanova et al., 2016; Clough et al., 2015). Published systematic reviews have evaluated the application of MSC-CM for the treatment of injuries and pathologies in several organs, such as acute renal failure, myocardial infarction, liver failure, lung disease, and nerve injury, where MSC-CM significantly promoted the repair and regeneration of tissue injuries and/or damaged organs (Muhammad et al., 2018; Akyurekli et al., 2015; JA, 2014). However, there have been no systematic reviews specifically evaluating the application of MSC-CM particularly in bone regeneration. Accordingly, the objective of this review was to assess the effectiveness of application of conditioned media derived from mesenchymal stem cells in bone regeneration in animal and human models.

## Methods

This systematic review was designed to answer the following question: What is the effectiveness of application of conditioned medium derived from mesenchymal stem cells in bone regeneration in animal and human models?

### Search strategy

We developed a search strategy to identify the studies published before February of 2018 in the electronic databases MEDLINE (OVID), EMBASE, CENTRAL (OVID) EBM Reviews - Cochrane Central Register of Controlled Trials, SCOPUS, Virtual Health Library (IBECS/LILACS/CUMED) using the following search terms: “Conditioned medium”, “Mesenchymal stem cell”, “Paracrine communication”, “Secretome”, “Tissue engineering”, “Regenerative medicine”, “Bone regeneration”, “Bone repair”, “Humans”, “Animal model”, “Experimental study”, “Clinical trial”, “Clinical study” and “Case reports”. The “snowballing technique” was also used as a search strategy in the lists of references of studies found in electronic databases. (See Appendix 1: Electronic search strategy).

### Study selection

The titles and abstracts of studies identified in the systematic search were evaluated independently by two researchers (MB and IL). Disagreements in the selection of articles were resolved by discussion and consensus. After the initial selection, potentially relevant articles were retrieved for a full-text assessment.

### Eligibility criteria

We included all experimental in vivo studies that evaluated bone regeneration after the application of MSC-CM in animal and human models reported in articles written in both English and Spanish with a publication date after 2000 and that reported measurable clinical, radiographic and/or histologic outcomes. We excluded studies that applied conditioned medium for regeneration of other tissues than bone, derived from other cell types, in vitro studies and review articles. (See Appendix 2: Exclusion criteria).

### Data extraction

After evaluation of full-text studies that met the inclusion criteria, we performed data extraction using a form developed for this review, where we obtained the following information: authors, publication year, objective, number (n) and population characteristics (age, sex, strain), methods and study design, intervention characteristics (cells used, preparation of conditioned medium, administration method, type of bone defect evaluated, duration of intervention, established groups (MSC-CM, comparison and control), outcomes assessed (bone regeneration, secondary outcomes, tests conducted for the measurement of results, most important results, statistical methods) and conclusions of the studies. Studies were grouped according to the following experimentation models: animal models and human models. (See Appendix 3: Data extraction form).

### Quality assessment

We assessed the risk of bias of animal studies with the SYstematic Review Centre for Laboratory animal Experimentation (SYRCLE) tool (Hooijmans et al., 2014), and of human studies with the Cochrane risk of bias tool (Higgins & Green, 2011). These risks of bias were classified into high, low or unclear. We used Revman 5.3 software to perform the graphic summary of risk of bias in the studies (Review Manager (RevMan) [Computer program], 2014). When studies were not experimental, we conducted a quality assessment with the “CARE checklist” tool for case reports (Gagnier et al., 2013).

### Intervention effect measure and synthesis of results

We performed a narrative description and an analysis of characteristics, findings and primary and secondary outcomes from the studies. Bone regeneration was reported in the original measures used in the studies. The results were treated with standardized mean difference (SSMD) due to the diversity in the measure instruments, measure units used, comparative interventions and the time when the effect was evaluated. These comparative measures were reported with their respective confidence intervals (CI) at 95%. Calculation were made by Revman 5.3 (Review Manager (RevMan) [Computer program], 2014). We explored statistical heterogeneity using I2 and Chi2 tests for bone regeneration outcome, we performed four forest plots, three of which were generated with a global diamond (meta-analysis). The studies were grouped according to the measure of the effect used and the time at which the evaluation of bone regeneration was performed. We assessed the percentage of bone regeneration at 2 and 4 weeks and the volume of bone regeneration at 8 weeks.

### Assessment of publication biases

Due to the limited number of studies included within the quantitative evidence of an overall effect of intervention on bone regeneration (meta-analysis), it was not possible to explore reporting bias using “funnel plots”.

### Assessment of the methodological quality of the systematic review

All phases of this systematic review were performed and reviewed according to PRISMA (Preferred Reporting Items for Systematic Reviews and Meta-Analyses) (Moher et al., 2009). (See Appendix 4**:** PRISMA checklist).

## Results

### Search results

The searches yielded a total of 6500 articles after removing duplicates. After the first screening, 6473 studies didn’t met the eligibility criteria, a total of 27 full-text articles were reviewed, from which 6 articles were excluded (Shang-Chun et al., 2016; Otsuru et al., 2018; Li et al., 2018; Byeon et al., 2010; Sakaguchi et al., 2017; Pethő et al., 2018). (See **section 2.3:** Eligibility criteria and Appendix 2: Exclusion criteria).

**Fig. 1:**
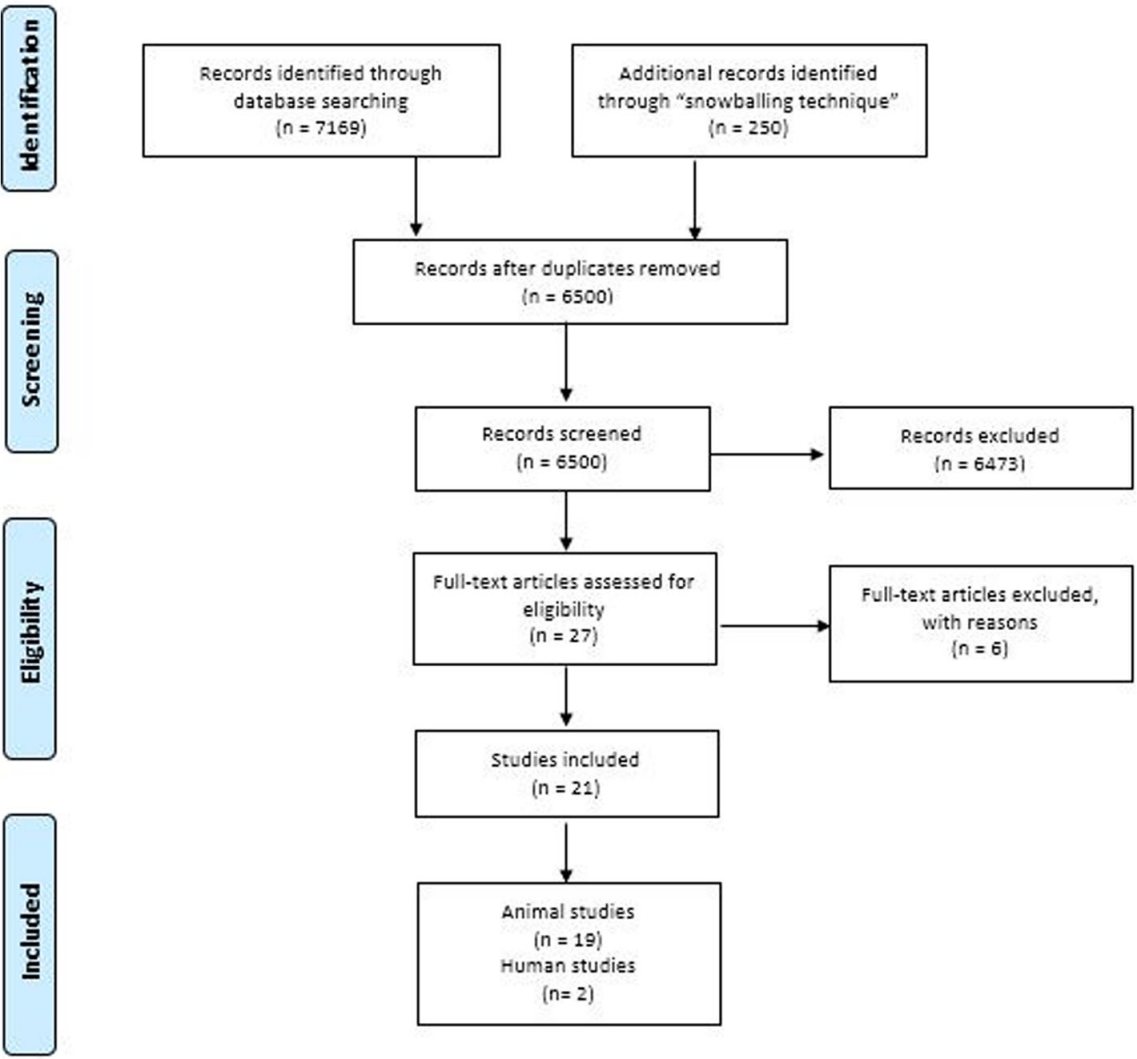
Flow Diagram of Systematic Search

We selected 21 articles that met the inclusion criteria, 19 of which described animal models and 2 of which described human models (a case report and a phase I clinical trial) (Fig. 1).

### Description of included studies

The first published study in humans is a case report (Katagiri, 2016) (Katagiri et al., 2016) that evaluated the safety and use of MSC-CM for alveolar bone regeneration in eight partially edentulous patients aged 45 to 67 years, which required bone augmentation, including maxillary sinus floor elevation (SFE), guided bone regeneration (GBR) and socket preservation (SP) for subsequent placement of dental implants. The second study was a phase I clinical trial (Katagiri, 2017ª) (Katagiri et al., 2017a) that evaluated the safety of use of the secretome of bone marrow-derived mesenchymal stem cells (MSC-CM) for surgical procedures of maxillary sinus floor elevation and bone grafting in 6 systemically healthy, partially edentulous patients.

We found 19 experimental studies using animal models where the conditioned media derived from mesenchymal stem cells were applied to regeneration of bone tissue. The characteristics of these studies are detailed in Table 1.

**Table 1:** Characteristics of studies

Author and year	Specie/ strain	Total n	Source of CM-Dosage	Application of MSC-CM
**(Ando et al.,**^2014^**)**	Mice / ICR	unclear	hMSCs − 20 μl	Distraction osteogenesis of tibia
**Chang et al.,**^2015^**)**	Rats/ Sprague-Dawley	21	rMSCs-1 ml	Circular calvarial bone defect of 5 mm diameter
**(Furuta et al.,**^2016^**)**	Mice/ (C57BL/6),CD9	77	hMSCs-100 μl	Femur fracture
**(Inukai et al.,**^2013^**)**	Dogs / hybrid	18	hMSCs-300 μl	Critical-size, box-type, one-wall intrabony defects (width 4 mm, height 5 mm)
**(Katagiri et al.,**^2013^**)**	Rats / Wistar/ST	24	hMSCs-NR	Circular calvarial bone defect of 5 mm diameter
**(Katagiri et al.,**^2015^**)**	Rabbits/ (JW/CSK)	15	hMSCs-NR	Bilateral cavity in the maxillary sinus (lateral window of 5 × 5 mm)
**(Katagiri et al.,**^2016^**)**	Humans	8	BM-hMSCs-3 ml	Maxillary sinus floor elevation (< 5 mm of residual bone)
**Katagiri et al.,**2017b**)**	Rats /Wistar/ST	24	hMSCs-NR	Circular calvarial bone defect of 5 mm diameter
**(Katagiri et al.,**2017a**)**	Humans	6	BM-hMSCs-3 ml	Maxillary sinus floor elevation (< 5 mm of residual bone)
**(Katagiri et al.,**2017c**)**	Rats / Wistar/ST	40	BM-hMSCs-30 μl	Circular calvarial bone defect of 5 mm diameter
**(Kawai et al.,**^2015^**)**	Rats /Wistar/ST	unclear	BM-hMSCs-30 μl	Periodontal defect of 1 mm diameter at palatal side of the first molar
**(Linero & Chaparro,**^2014^**)**	Rabbits / New Zealand	19	Ad-hMSCs-16,6 μl	Bilateral mandibular bone defects of 10 mm diameter
**(Ogata et al.,**^2015^**)**	Rats / Wistar/ ST	24	hMSCs-1 ml	Exposed bone after tooth extraction in rats with BRONJ
**(Osugi et al.,**^2012^**)**	Ratas Wistar/ST	40	BM-hMSCs-6 ml	Circular calvarial bone defect of 5 mm diameter
**(Qin et al.,**^2016^**)**	Rats / Sprague Dawley (SD)	6	BM-hMSCs (Evs) -100 μg	Circular calvarial bone defect of 5 mm diameter
**(Sanchooli et al.,**^2017^**)**	Rats / Wistar/ST	24	Ad-rMSCs-NR	Circular calvarial bone defect of 5 mm diameter in rats with hypothyroidism
**(Tsuchiya et al.,**^2013^**)**	Rats /Sprague- Dawley	15	BM-rMSCs-1 mg/ml	Implant in insertion socket of 1,5 mm diameter created on the femur
**(Tsuchiya et al.,**^2015^**)**	Rats / Sprague-Dawley	24	BM-rMSCs-NR	Circular calvarial bone defect of 8 mm diameter
**(Wang et al.,**2012b**)**	Rats /Sprague–Dawley (SD)	unclear	hMSCs-100 μl	Fracture - Bone defect of 2 mm in the middle third of the fibula in rats with diabetes
**(Wang et al.,**^2015^**)**	Rats / Sprague-Dawley (SD)	8	hUCMSCs-10 μg	Circular calvarial bone defect of 5 mm diameter
**(Xu et al.,**^2016^**)**	Rats / SD	24	F-hMSCs-100 μl	Distraction osteogenesis of tibia transverse osteotomy

*hMSCS* Human mesenchymal stem cells, *rMSCS* Rat mesenchymal stem cells, *BM* Bone marrow, *Ad* adipose tissue, *hUCMSCs* Mesenchymal stem cells derived from human umbilical cord, *F-hMSCs* human fetal mesenchymal stem cells. *Evs* Extracellular vesicles. *NR* No report

### Description of intervention

#### Conditioned medium sources

Conditioned media used in the studies were obtained from MSC of different tissues. In 81% (17 studies), human MSCs were used, and in 19% (4 studies), MSCs of animal origin were used, specifically those of rats (Chang et al., 2015; Sanchooli et al., 2017; Tsuchiya et al., 2013; Tsuchiya et al., 2015). In the studies that used conditioned media of human origin, 6 reported that MSCs were derived from bone marrow (Katagiri et al., 2016; Katagiri et al., 2017a; Katagiri et al., 2017c; Kawai et al., 2015; Osugi et al., 2012; Qin et al., 2016), one from adipose tissue (Linero & Chaparro, 2014), another from umbilical cord (Wang et al., 2015) and one more used fetal human MSC (Xu et al., 2016), while the remaining 8 studies did not specify the human MSC tissue of origin (Ando et al., 2014; Furuta et al., 2016; Inukai et al., 2013; Katagiri et al., 2013; Katagiri et al., 2015; Katagiri et al., 2017b; Ogata et al., 2015; Wang et al., 2012b). Of the studies that used animal MSCs to obtain conditioned media, 2 reported that MSCs were derived from bone marrow (Tsuchiya et al., 2013; Tsuchiya et al., 2015) and one used MSCs derived from adipose tissue (Sanchooli et al., 2017).

#### Application of MSC-CM

In the human models: Katagiri, 2016 (Katagiri et al., 2016) performed procedures of maxillary sinus floor elevation and guided bone regeneration with an implant of beta-tricalcium phosphate (B-TCP) soaked in MSC-CM and socket preservation with an implant of atelocollagen sponge soaked in MSC-CM. Katagiri, 2017ª (Katagiri et al., 2017a) also performed maxillary sinus floor elevation procedures with an implant of B-TCP soaked in MSC-CM, and in the control group, B-TCP without MSC-CM was implanted.

In the animal models: In ten studies, MSC-CM were applied to circular bone defects, eight of which applied the MSC-CM in calvarial bone defects of 5 mm in diameter (Chang et al., 2015; Katagiri et al., 2013; Katagiri et al., 2017b; Katagiri et al., 2017c; Osugi et al., 2012; Qin et al., 2016; Sanchooli et al., 2017; Wang et al., 2015), one in calvarial bone defects of 8 mm in diameter (Wang et al., 2012b), and another in bilateral bone defects of 10 mm in diameter in the mandibular angles of rabbits (Linero & Chaparro, 2014). In two studies, MSC-CM were applied in periodontal bone defects (Inukai et al., 2013; Kawai et al., 2015). In one study MSC-CM was applied in maxillary sinus floor elevation procedures (Katagiri et al., 2015). In two studies, MSC-CM was applied in models of distraction osteogenesis of tibia (Ando et al., 2014; Xu et al., 2016). In two more studies MSC-CM was evaluated in fractures, one in a femur (Furuta et al., 2016) and the other in the middle third of the fibula in rats with diabetes (Wang et al., 2012b). One study evaluated osseointegration of an implant soaked in MSC-CM in a socket created in a femur (Tsuchiya et al., 2013), and another study evaluated the therapeutic effects of MSC-CM in a bisphosphonate-related osteonecrosis of the jaw (BRONJ) model in rats (Ogata et al., 2015) (Table 1).

#### Comparison and control groups

Most studies compared the application of the MSC-CM with different treatments, demonstrating more than one comparison and/or control group. In 11 studies, applications of MSC-CM and phosphate buffered saline (PBS) were compared (Inukai et al., 2013; Katagiri et al., 2013; Katagiri et al., 2015; Katagiri et al., 2017b; Katagiri et al., 2017c; Kawai et al., 2015; Osugi et al., 2012; Tsuchiya et al., 2013; Tsuchiya et al., 2015; Wang et al., 2015; Xu et al., 2016). In 10 studies, the control defects were allowed to heal by second intention, leaving the bone defects without filling (Furuta et al., 2016; Inukai et al., 2013; Katagiri et al., 2013; Katagiri et al., 2017b; Katagiri et al., 2017c; Kawai et al., 2015; Ogata et al., 2015; Osugi et al., 2012; Sanchooli et al., 2017; Wang et al., 2012b). In 4 studies, a comparison was with application of the MSC (Linero & Chaparro, 2014; Osugi et al., 2012; Sanchooli et al., 2017; Wang et al., 2015), another 5 studies used Dulbecco’s Modified Eagle Medium (DMEM) for a control group (Ando et al., 2014; Ogata et al., 2015; Osugi et al., 2012; Tsuchiya et al., 2013; Wang et al., 2012b), and in 5 studies, different scaffolds or media (collagen gel, hydrogel, blood plasma hydrogel, or serum-free medium) were used (Linero & Chaparro, 2014; Chang et al., 2015; Qin et al., 2016; Sanchooli et al., 2017; Xu et al., 2016) (Table 2).

**Table 2:** Results of studies

Author and year	Outcome	MSC-CM (n)	Comparison(n)	Control (n)	Time	Conclusions
**(Ando et al.,**^2014^**)**	% of new bone callus in the distraction gap	MSC-CM 62% (10)	FB-CM: 37%(10)	DMEM: 32% (10)	15 days	MSC-CM accelerates the formation of new bone callus, shortening the period required for DO treatment
**(Chang et al.,**^2015^**)**	% of new bone formation over the total area of the defect	HCM:NC	NCM: NC	__	56 days	Bone repair is significantly increased with hypoxic MSC-CM by enhancement of endogenous MSCs migration and adhesion and gene regulation by miRNA
**(Furuta et al.,**^2016^**)**	Bone union presence of bridging callus on two cortices	MSC-CM (9): NC	Exosomes (9): NC	PBS (15): NC	6 weeks	MSC-derived exosomes rescued the retardation of fracture healing in CD9 −/− mice
**(Inukai et al.,**^2013^**)**	Bone regeneration area	MSC-CM /Scaffold: 4.89 ± 1.08 mm2 (6)	PBS/ Scaffold: 2,4 mm2 (6)	No implant/Scaffold: 1,8 mm2 (6)	4 weeks	Large amount of bone and cement formation was observed in the MSC-CM group. There was minimal inflammatory cell infiltration in the MSC-CM
**(Katagiri et al.,**^2013^**)**	% area of newly regenerated bone over the total area of the defect	MSC-CM (81.50% + −2.7%), (93.07% + − 6.6%) (4)	PBS (60.63% + − 5.8%) (84.04% + − 4.9%) (4)	Defect (unfilled) (8.63% + − 1.78%) (4)	2 y 4 weeks	MSC-CM group showed higher new bone regeneration compared with control groups, at 4 weeks the defect was completely replaced by mature bone tissue
**(Katagiri et al.,**^2015^**)**	% of newly formed bone area in the elevated sinus floor	MSC-CM/ B-TCP Aprox. 15%, 22%, 37% (NC)	PBS/B-TCP Aprox. 9%,17%, 35% (NC)	__	2, 4 y 8 weeks	Sinus floor elevation with MSC-CM/B-TCP enhanced early bone regeneration compared to B-TCP alone
**(Katagiri et al.,**^2016^**)**	New formed bone in augmented area	MSC-CM/B-TCP: NR	B-TCP: NR	__	8–9 months	MSC-CM promoted early bone formation and mineralization compared to B-TCP without MSC-CM, No bone resorption was observed
**(Katagiri et al.,**2017b**)**	% area of newly formed bone over the total area of the defect	MSC-CM (72.3 ± 17.1%) (24)	PBS: (30.9 ± 6.2%) (24)	Defect (unfilled)(22.2 ± 8.0%) (24)	2 weeks	MSC-CM enhanced the migration of endogenous cells, which enabled the formation of more blood vessels and bone tissue in the bone defect
**(Katagiri et al.,**2017a**)**	New formed bone area in maxillary sinus floor elevation.	MSC-CM/B-TCP (4) NR	B-TCP (2) NR	__	6 months	MSC-CM was used safely and enhanced vascularization and early bone formation in maxillary SFE
**(Katagiri et al.,**2017c**)**	% of the area of newly formed bone over the total area of the defect	MSC-CM (74.94 ± 19.11%) (8)	PBS: (31.61 ± 5.23%) (8)	Defect (unfilled)(15.27 ± 8.21%) (8)	2 weeks	A higher percentage of bone formation was observed in CM and CC groups, in comparison with the other groups. MSC-CM elicit osteogenesis and angiogenesis
**(Kawai et al.,**^2015^**)**	Qualitative description of histological findings	MSC-CM: NR	PBS: NR	Defect (unfilled) NR	2 y 4 weeks	MSC-CM promoted periodontal tissue regeneration through mobilization of endogenous MSCs, angiogenesis and differentiation
**(Linero & Chaparro,**^2014^**)**	% of regenerated bone tissue, compared to the initial defect.	AdMSC-CM: 75% (3)	Ad-MSC/HBPH: 62% (4)	Blood plasma hydrogel: 32% (4)	45 days	Ad-MSC improves bone regeneration, and the quantity and quality of regenerated bone is similar with paracrine factors collected and applied as CM instead of Ad-MSCs
**(Ogata et al.,**^2015^**)**	Volume of bone sequestra (mm3)	MSC-CM Aprox (0.4 mm3) (8)	DMEM: Aprox (2.6 mm3) (8)	Non treatment: Aprox (2.8 mm3) (8)	2 weeks	Open alveolar sockets in 63% of the rats with BRONJ healed with complete soft tissue coverage, whereas the exposed necrotic bone remained in the other groups
**(Osugi et al.,**^2012^**)**	% area of newly formed bone over the total area of the defect	MSC-CM (49.5% + − 2.7%), (64.4% + − 19.7%)(4)	PBS:(24.9% + − 2.2%) (36.1% + −2.9%) (4)	Defect (unfilled) (23.4% + − 4.5%) (28.6% + − 5.3%) (4)	4 y 8 weeks	The area of ​​new regenerated bone was significantly higher in the MSC-CM group compared to the other groups.
**(Qin et al.,**^2016^**)**	Volume of regenerated bone (mm3)	Evs-MSC: 4.0 ± 1.9 mm3 (6)	Hydrogel 1.3 ± 0.7 mm3 (6)	__	8 weeks	The Evs derived from human BMSCs contained in gel, accelerated bone regeneration and showed a clear increase in the repair of the defect.
**(Sanchooli et al.,**^2017^**)**	New bone volume (mm3)	MSC-CM (2.126 + − 0.064) (6), (3.113 + − 0.021 mm3) (6)	Collagen gel (1.433 + − 0.266), (2.536 + − 0.085 mm3) (6)	Empty defect (0.173 + − 0.060), (0.626 + − 0.104 mm3) (6)	4 y 8 weeks	Significantly greater bone volume was observed in the AdMSC-CM group compared with the other groups
**(Tsuchiya et al.,**^2013^**)**	% direct implant- bone contact / peri-implant length.	MSC-CM (74.3 + − 2.8)(5), (84.7 + − 5.4) (5)	PBS: (63.7 + − 5.8) (5) (82.3 + − 2.4) (5)	DMEM: (62.3 + − 5) (5), (81.6 + − 4) (5)	7 y 28 days	The removal torque increased gradually over time in the CM group. CM promoted integration into bone during an early stage.
**(Tsuchiya et al.,**^2015^**)**	% newly formed bone area	CM 14.5% (3), 24.1% (3)	CM-HM 22.7%, 26.9% (3)	PBS: 8.1%, 15.8% (3)	4 y 8 weeks	Bone formation was increased in the CM and CM-HM groups, compared with the other groups.
**(Wang et al.,**2012b**)**	Bone volume, healing rate of the fracture.	MSC-CM (6,6 mm3) 36,8% (19)	DM- MEM: (1,7 mm3), 0% (10)	Unfilled: (2,5 mm3) 0% (10)	8 weeks	MSC-CM promoted angiogenesis and fracture healing in a diabetic model. Enhanced bone ingrowth and fracture healing rates compared to the other groups.
**(Wang et al.,**^2015^**)**	Ratio of bone volume / total volume	MSC-CM: Aprox 0.04 (4), 0.07 (4)	PBS: 0.02 (4), 0.04 (4)	__	4 y 8 weeks	Bone generatioserum was increased in the group of factors secreted by hUCMSCs than in the control group
**(Xu et al.,**^2016^**)**	Bone volume / total tissue volume	Secretome: NC	PBS: NC	Serum-free medium: NC	6 weeks	The secretome increased the osteogenic differentiation potential of the rBMSCs and accelerated bone healing and bone consolidation during distraction osteogenesis.

*NC* Results not clear, approximate values according to graphs; *NR* It does not report the results. *CC* Cytokine cocktail. *MSC-CM* Mesenchymal stem cells- Conditioned medium. *FB-CM* Fibroblasts conditioned medium. *DMEM* Dulbecco’s modified Eagle’s medium. *DO* Distraction osteogenesis. *HCM* Hypoxic conditioned medium. *NCM* Normoxic conditioned medium. *PBS* Phosphate-Buffered Saline, *B-TCP* Beta–tricalcium phosphate scaffolds. *HBPH* Human blood plasma hydorgels. *Evs* Extracellular vesicles. *CM-HM* Conditioned medium- hydrophilic membrane. *hUCMSCs* Mesenchymal stem cells derived from human umbilical cord

### Risk of bias of studies

**Fig. 2:**
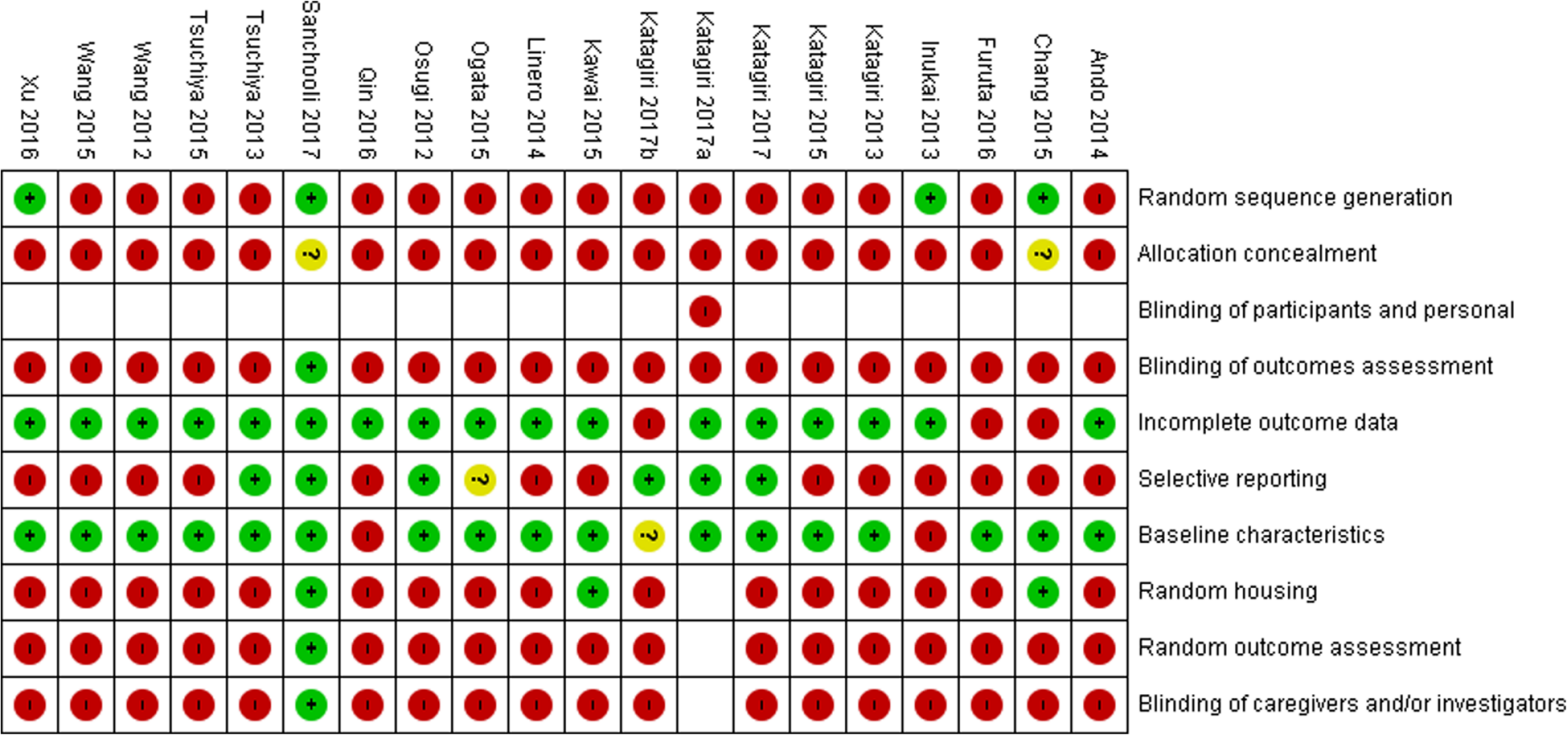
Risk of bias in individual studies. Author name and year of publication of each study with their respective result in each item of assessment**.** Negative sign (−) indicates a high risk, a Question mark (?) indicates not clear risk, a Positive sign (+) indicates low risk of bias

In 19 studies (95%) that underwent the assessment, a high risk of bias was observed for most parameters. Only one study scored a low risk of bias in 8 of the 9 parameters evaluated (Sanchooli et al., 2017) (Fig. 2).

### Description of a primary outcome: bone regeneration

#### Instruments and evaluation measures

In studies performed in humans (Katagiri et al., 2016; Katagiri et al., 2017a), bone regeneration was evaluated by measuring the area of newly formed bone using panoramic radiographs and computerized tomography (CT) before and after maxillary sinus floor elevation procedures. In addition, histological analyses of bone biopsies taken out of the regenerated bone were performed 6 months after the surgical procedure.

In animal model studies, different tools were used for the measurement and analysis of bone regeneration. Fifteen studies used X-rays and/or microcomputerized tomography (micro-CT) (Linero & Chaparro, 2014; Chang et al., 2015; Furuta et al., 2016; Inukai et al., 2013; Katagiri et al., 2013; Katagiri et al., 2017b; Katagiri et al., 2017c; Ogata et al., 2015; Osugi et al., 2012; Qin et al., 2016; Tsuchiya et al., 2013; Tsuchiya et al., 2015; Wang et al., 2012b; Wang et al., 2015; Xu et al., 2016), 18 studies performed histological and/or morphometric analyses (Linero & Chaparro, 2014; Ando et al., 2014; Chang et al., 2015; Inukai et al., 2013; Katagiri et al., 2013; Katagiri et al., 2015; Katagiri et al., 2017b; Katagiri et al., 2017c; Kawai et al., 2015; Ogata et al., 2015; Osugi et al., 2012; Qin et al., 2016; Sanchooli et al., 2017; Tsuchiya et al., 2013; Tsuchiya et al., 2015; Wang et al., 2012b; Wang et al., 2015; Xu et al., 2016), and one study performed a stereological analysis (measure of the volume of a new bone and connective tissue) and an enumeration of bone cells (Sanchooli et al., 2017). In addition, 7 studies conducted an immunohistochemistry analysis (Katagiri et al., 2015; Katagiri et al., 2017b; Katagiri et al., 2017c; Kawai et al., 2015; Ogata et al., 2015; Wang et al., 2015; Xu et al., 2016) to assess the presence of MSC at the defect site or regenerated area and one study assessed the osseointegration of an implant with a removal torque test (Tsuchiya et al., 2013). Another study conducted a mechanical test in a model of distraction osteogenesis (Xu et al., 2016).

The bone regeneration was reported in terms of the percentage area of newly formed bone over the total area of bone defect (9 studies) (Linero & Chaparro, 2014; Ando et al., 2014; Chang et al., 2015; Katagiri et al., 2013; Katagiri et al., 2015; Katagiri et al., 2017b; Katagiri et al., 2017c; Osugi et al., 2012; Tsuchiya et al., 2015), the volume of new regenerated bone relative to the total volume of bone defect (3 studies) (Qin et al., 2016; Sanchooli et al., 2017; Wang et al., 2012b), the ratio of bone volume over the volume of tissue (one study) (Xu et al., 2016), the fractions of area and volume of newly formed bone tissue (2 studies) (Inukai et al., 2013; Wang et al., 2015), the volume of the sequestra in the model of bisphosphonate-related osteonecrosis of the jaw (one study) (Ogata et al., 2015), the osseointegration of the implant measured as the rate of bone contact (one study) (Tsuchiya et al., 2013) and the presence of bridging callus on two cortices in fractures (one study) (Furuta et al., 2016). Only one study reported bone regeneration in a qualitative manner by using the findings of histological analyses (Kawai et al., 2015) (Table 2).

#### Effect of MSC-CM on bone regeneration

In the studies performed in human models (Katagiri et al., 2016; Katagiri et al., 2017a), the radiographic images and CT showed an early mineralization of regenerated bone without bone resorption or evident inflammation of maxillary sinus membrane. Histological analysis showed increased formation of new bone tissue as well as increased vascularity of regenerated area with little infiltration of inflammatory cells in comparison with the control cases, where the formation of new bone was significantly lower and there was a greater inflammatory infiltrate.

In general, studies in animal models reported that application of MSC-CM to bone defects significantly increased regeneration of bone tissue in comparison with other intervention or control groups (Linero & Chaparro, 2014; Chang et al., 2015; Furuta et al., 2016; Inukai et al., 2013; Katagiri et al., 2013; Katagiri et al., 2017b; Katagiri et al., 2017c; Kawai et al., 2015; Osugi et al., 2012; Qin et al., 2016; Sanchooli et al., 2017; Tsuchiya et al., 2015; Wang et al., 2012b; Wang et al., 2015). Studies that carried out maxillary sinus floor elevation reported early mineralization in the grafted area upon application of conditioned media (Katagiri et al., 2015). The studies that evaluated bone regeneration during distraction osteogenesis reported that the secretome of MSCs accelerated the formation of new bone callus and bone healing, shortening the period required for treatment (Ando et al., 2014; Xu et al., 2016). In models of bone fracture, conditioned medium helped to improve new bone formation, angiogenesis and consolidation of the fracture (Furuta et al., 2016; Wang et al., 2012b). The studies that evaluated bone regeneration in periodontal defects, reported that application of conditioned medium promoted differentiation of stem cells to osteoblastic lineage, endogenous cellular migration and bone regeneration, showing a large amount of new bone and cement formation and minimal infiltration of inflammatory cells (Inukai et al., 2013; Kawai et al., 2015). In the study that evaluated therapeutic effects of MSC-CM in rats with bisphosphonate-related osteonecrosis of the jaw (BRONJ), 63% of the open sockets healed with full coverage by soft tissue (Ogata et al., 2015). In the study that evaluated implant osseointegration, it was reported that the removal torque was significantly higher in the group where the MSC-CM were applied, than in control groups (Tsuchiya et al., 2013) (Table 2).

Of the 21 articles included, 5 did not report the volume of CM used and of the remaining 17, only 4 report the protein concentration (Linero & Chaparro, 2014; Tsuchiya et al., 2013; Wang et al., 2015; Xu et al., 2016). The non-reporting of MSC-CM dose used, the variability in the amount of CM applied, but above all, not identifying the concentration of proteins contained in the applied conditioned medium, prevent a comparative analysis of the studies and therefore to find a relation between the dose of MSC-CM and therapeutic effectiveness.

In all the studies that performed histological analysis, it was observed that in bone defects treated with MSC-CM there was a greater formation of new, primarily mineralized, regenerated bone with little or no infiltration of inflammatory cells, whereas the control groups showed reduced formation of bone tissue with less mineralization, greater amounts of connective tissue and increased infiltration of inflammatory cells (Linero & Chaparro, 2014; Ando et al., 2014; Inukai et al., 2013; Katagiri et al., 2013; Katagiri et al., 2015; Katagiri et al., 2017b; Katagiri et al., 2017c; Kawai et al., 2015; Osugi et al., 2012; Qin et al., 2016; Tsuchiya et al., 2015; Wang et al., 2012b; Xu et al., 2016).

Most studies reported that cytokines and growth factors contained in conditioned medium act synergistically to stimulate the migration and proliferation of osteoprogenitor cells, promote osteogenesis and bone regeneration and improve the early vascularization (Linero & Chaparro, 2014; Chang et al., 2015; Kawai et al., 2015). MSC-CM contains a mixture of multiple growth factors at relatively low concentrations that promote bone regeneration without causing a severe inflammatory response (Katagiri et al., 2016; Katagiri et al., 2017a; Ando et al., 2014; Chang et al., 2015; Furuta et al., 2016).

#### Meta-analysis of MSC-CM effect on bone regeneration

We grouped 7 studies that shared similar characteristics to evaluate through a meta-analysis of the effect of MSC-CM on bone regeneration in terms of the amount of newly formed tissue and time of tissue regeneration.

**Fig. 3:**
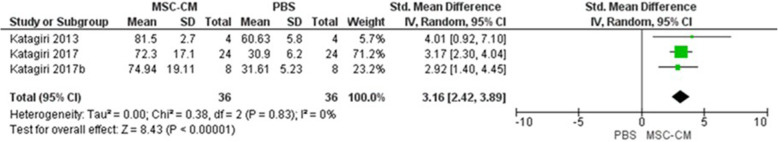
Percentage of bone regeneration at 2 weeks. MSC-CM vs. PBS. Details the effect of the application of MSC-CM compared with PBS control at 2 weeks

**Fig. 4:**
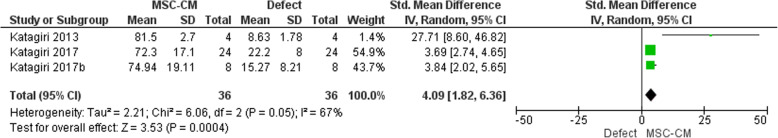
Percentage of bone regeneration at 2 weeks. MSC-CM vs. Defect (unfilled). Compares the effect of MSC-CM vs. an empty defect at 2 weeks

**(**Fig. 3**)** details the effect of the application of MSC-CM compared with PBS control at 2 weeks (Katagiri et al., 2013; Katagiri et al., 2017b; Katagiri et al., 2017c). We observed an overall favorable effect of MSC-CM with **SMD: 3.16** (95% CI 2.42, 3.49), which indicates a significant difference between the MSC-CM and PBS groups. Regarding the comparison of MSC-CM with an empty defect, the favorable MSC-CM effect was maintained (**SMD 4.09**, 95% CI 1.82 to 6.36), indicating a significant difference between the MSC-CM and empty defect groups (Katagiri et al., 2013; Katagiri et al., 2017b; Katagiri et al., 2017c) (Fig. 4).

**Fig. 5:**
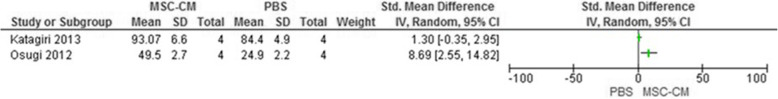
Percentage of bone regeneration at 4 weeks. MSC-CM vs. PBS. Details the effect of the application of MSC-CM compared with PBS at 4 weeks

At 4 weeks of bone regeneration, (Fig. 5**)** details the effect of the application of MSC-CM compared with PBS. In Osugi 2012 (Osugi et al., 2012), there was a favorable MSC-CM effect with a statistically significant difference (**SMD: 8.69**, 95% CI 2.55, 14.82); Katagiri, 2013 (Katagiri et al., 2013) also presented a favorable MSC-CM effect; however, the difference between the two interventions was not significant (**SMD: 1.30**, 95% CI -0.35, 2.95). Due to high heterogeneity (I2: 81%), it was not possible to obtain the overall effect of the intervention.

**Fig. 6:**
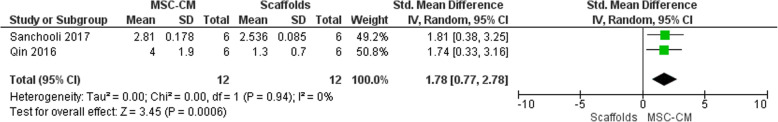
Volume of bone regeneration at 8 weeks. MSC-CM vs. Scaffolds. Details the effect of the application of MSC-CM compared with scaffolds at 8 weeks

**(**Fig. 6**)** details the results of analysis of the overall effect of the intervention after the application of MSC-CM compared with the implantation of scaffolds (gel of type 1 collagen, Hydrogel) as measured by the volume of regenerated bone at 8 weeks (Qin et al., 2016; Sanchooli et al., 2017) when the favorable MSC-CM effect was maintained with statistically significant differences (**SMD: 1.78**, 95% CI 0.77, 2.78).

### Secondary outcomes

#### Markers and gene expression

Several studies evaluated the expression of osteogenic and angiogenic genes and markers in MSCs after MSC-CM application by using RT-PCR analysis, reporting an increase in the expression levels of ALP, Col I*a*2, OCN, Runx2, GAPDH, VEGF-A, ANG-1 and ANG-2 in MSCs. This indicated that MSC-CMs promote osteoblast differentiation, migration of endogenous MSCs and angiogenesis (Chang et al., 2015; Katagiri et al., 2013; Katagiri et al., 2017c; Kawai et al., 2015; Osugi et al., 2012; Qin et al., 2016; Tsuchiya et al., 2013; Wang et al., 2015; Xu et al., 2016).

#### Inflammatory response

In the human studies (Katagiri et al., 2016; Katagiri et al., 2017a), the clinical observations and blood tests showed no abnormal findings except for lesser signs of inflammation after surgery. No local or systemic alterations were observed, and no patient showed abnormal swelling or delayed healing.

Animal studies reported that there was no inflammatory response to the application of MSC-CM, and histological analyses showed reduced infiltration of inflammatory cells in MSC-CM groups in comparison with other groups (Inukai et al., 2013; Katagiri et al., 2013; Katagiri et al., 2017c; Kawai et al., 2015; Osugi et al., 2012; Xu et al., 2016).

#### Angiogenesis

In Katagiri, 2017 (Katagiri et al., 2017b), the results indicated that the presence of vascular endothelial growth factor (VEGF) in MSC-CM promoted the migration of endothelial cells and endogenous stem cells, which allowed the formation of more blood vessels and bone tissue in the defect. They also observed that neutralization of VEGF in MSC-CM abolished angiogenesis, which caused only a minor migration of endogenous stem cells into the defect and reduced new bone formation. Similarly, Kawai, 2015 (Kawai et al., 2015) demonstrated that MSC-CM strongly promoted angiogenesis by increasing the expression levels of angiogenic markers, such as VEGF-A, ANG-1 and ANG-2, in MSCs cultured with MSC-CM. In addition, the results in Osugi 2012 (Osugi et al., 2012) indicated that MSC-CMs have the potential to mobilize endogenous MSCs to promote angiogenesis and bone regeneration.

#### Antiresorptive activity

In Ogata 2015 (Ogata et al., 2015), the application of MSC-CM in rats with induced BRONJ generated an effect of maintaining the osteoclast function. The results indicated that 63% of rats with BRONJ in the MSC-CM group healed with a full coverage of connective tissue, while in the control group, exposed necrotic bone and inflamed soft tissue were observed. The anti-apoptotic, anti-inflammatory and angiogeneic effects of MSC-CM dramatically regulated the turnover of local bone, generating positive results in the treatment of BRONJ.

## Discussion

Bone regeneration is a physiological process that requires the migration and proliferation of specific cells in a biological substrate of soluble factors and proteins, which coordinate the formation of new tissue, thus restoring bone structure and function. Local unfavorable conditions, such as inadequate blood supply, damage to the surrounding soft tissues, mechanical instability, extensive loss of bone tissue and local infection, cause a delay in the repair process and persistence of bone defects (Rosset et al., 2014). Although the exact mechanisms that regulate the process of bone regeneration at the molecular level are not yet fully understood (Dimitriou et al., 2011), several methods have been proposed for bone reconstruction, ranging from autografts, allografts, xenografts and bone substitutes (Pilipchuk et al., 2015). These treatment strategies have several drawbacks. An autologous bone graft is widely used for its osteoinductive, osteoconductive and osteogenic properties and immunogenic compatibility, but this implies the need for a double surgical procedure, which causes morbidity at the donor site, thus making it difficult to use (Goulet et al., 1997); in addition, the absence of cell populations in allografts and xenografts results in poor osteogenic and osteoinductive properties (Padial et al., 2015). To overcome these limitations, regenerative medicine aims to replace or regenerate tissues or organs to restore and stabilize their normal functions (Mason & Dunnill, 2008) using different tools, such as tissue engineering, gene therapy, cell therapy and therapy based on growth factors. An interest in cell therapy and specifically in mesenchymal stem cells and their clinical application has grown exponentially in the past 25 years (Le Blanc & Davies, 2018). MSCs are relatively easy to harvest and expand ex vivo, are able to modulate the immune system, and are able to repair injured tissues in particular; therefore, MSCs have become an attractive source for many applications in regenerative medicine (Le Blanc & Davies, 2018; Klyushnenkova et al., 2005; Caplan & Correa, 2011). Several studies showed the beneficial effects of stem cell therapy in diseases such as osteoarthritis (Yang et al., 2015), acute myocardial infarction (Zhang et al., 2013), wound healing (Yoshikawa et al., 2008), kidney damage (Ma et al., 2013), peripheral nerve injury (Wang et al., 2009), bone defects, and others (Linero & Chaparro, 2014). Thanks to the large amount of scientific research on in vitro and in vivo models and 799 clinical trials reported by the US National Institutes of Health (NIH) (clinical trials.gov) (consultation carried out in June 2018), we know that MSC therapy is a safe and effective method for treatment of certain diseases and/or conditions. Originally, it was hypothesized that due to their proliferative and multipotent capacities, transplanted stem cells differentiated into the cells of interest and replaced the injured tissue (Spees & Lee, 2016; Ankrum & Karp, 2010); however, the results of animal studies and clinical trials have demonstrated that a curative effect can be attributed to their ability to secrete growth factors, cytokines, chemokines, and extracellular matrix molecules at the receptor site, which modulate endogenous cell migration and stimulate angiogenesis and differentiation of the patient’s stem cells, thus inducing the formation of new tissues (Muhammad et al., 2018; Chen et al., 2008). The secreted factors are referred to as the secretome and can be found in the environment where mesenchymal stem cells grow; that is, mesenchymal stem cell-conditioned medium (MSC-CM). MSC-CM contains the regenerative agents capable of promoting and modulating the formation of new tissues (Muhammad et al., 2018; JA, 2014). The application of MSC-CM has been shown to be effective in diseases such as focal cerebral ischemia (Inoue et al., 2013), Alzheimer’s disease (Mita et al., 2015), acute renal failure (Matsushita et al., 2017), rheumatoid arthritis (Ishikawa et al., 2016), diabetes (Izumoto-Akita et al., 2015), and other diseases (Muhammad et al., 2018; Akyurekli et al., 2015; Shimojima et al., 2016; Yamaguchi et al., 2015; Wakayama et al., 2015; Fukuoka & Suga, 2015) and in conditions that affect the bone tissue, such as nonunion fractures and bone defects (Linero & Chaparro, 2014; Shang-Chun et al., 2016; Otsuru et al., 2018).

Molecular mechanisms and key factors involved in the therapeutic effects of MSC secretome are still unknown (Bari et al., 2019). Some studies have compared the biological effects of secretome with those of stem cells and in general terms, most of them have shown that the secretoma has greater or equal efficacy to that of cells (L et al., 2019; Tran & Damaseer, 2015). Porzionato and collaborators, demonstrated in a model of bronchopulmonary dysplasia, that the extracellular vesicles contained in the MSC secretome obtained better results in terms of pulmonary vascularity and alveolarization with respect to MSC (Porzionato et al., 2019); Sang Mook Lee, et al.; found no significant differences in the potential to induce immune tolerance in the animals to which MSC vs MC-MSC were applied in an allogeneic mouse skin transplant model (Lee et al., 2014), likewise, our research group in a previous study, in a rabbit model where bicortical mandibular bone defects were performed, we found that the amount of neoformed bone tissue, bone density, the arrangement of collagen fibers, maturation and calcification of the inorganic matrix, were very similar on the side treated with MSC vs the side treated with the MC-MSC, demonstrating morphologically, radiologically and histologically, that there are no significant differences between the transplantation of MSC and the application MSC-CM in bone regeneration (Linero & Chaparro, 2014). Three other articles included in this review compared the application of MSC vs MSC-CM for bone regeneration. Which reported that although increased bone regeneration was observed in all groups where MSC-CM were applied, the difference with the MSC groups was not significant (Ando et al., 2014; Osugi et al., 2012; Sanchooli et al., 2017). The therapeutic differences between the application of cells and conditioned medium, perhaps arises from the possibility of using a cell-free product, which offers advantages over cell therapy. Although it has been reported that the application of MSC is safe, using only the proteins they secrete and not the cells, avoids the risk of emboli formation after intravenous administration and decreases the risk of pathological and tumorogenic transformation due to uncontrolled cellular differentiation (Bari et al., 2019). In addition, the application of cells is subject to problems such as poor cellular survival in the host after transplantation, poor ability to differentiate from transplanted cells, sequestration at non-target sites and failure of cells to graft in the long term (L et al., 2019). Secretome preserves the composition of the parental cells while maintaining the same privileged immunity of the MSC, allowing its allogeneic application without immune activation. Conditioned medium can be manipulated, stored and characterized more easily than cells, sterilization is possible without loss of efficacy, and they are ready for immediate use.

In this review, we systematically collected all the available data in the literature and critically evaluated whether the conditioned medium derived from MSC significantly promoted bone regeneration in animals and humans, making an objective and clear assessment of the scientific evidence published, resulting in a systematic review developed specifically to evaluate the effect of MSC-CM on regeneration of bone tissue.

The results of this systematic review indicate that research on this topic has been conducted mainly in animals. Critical evaluation of interventions in this type of models is a real challenge, since the reporting of methodological aspects and results is generally poor, the random allocation of animals into experimental and control groups is not a standard practice, the sample size is relatively small, and several details of the experimental designs were not included in the publications. For this reason, it is important to assess the similarity in the base characteristics between the control group and the experimental group as a necessary parameter (Hooijmans et al., 2014). In the assessment of risk of bias in the articles included in this review, we observed that 95% of these studies presented a high risk of bias in most parameters, mainly due to not reporting randomization in the selection of the animals, concealment of sequence and blinding of the evaluators. This observation applies to many animal studies published at the global level, since most of them have a high risk of bias for the abovementioned aspects (Hirst et al., 2014). Currently, the “SYRCLE’s RoB” tool, available from the year 2014, which was developed to establish consistency in the assessment of risk of bias of systematic reviews carried out with animal studies, facilitates the critical evaluation of the evidence and improves the abilities of these studies to transfer to human models (Hooijmans et al., 2014). It is worth noting that one of the studies included, which was published in 2017 (Sanchooli et al., 2017), presented a low risk of bias in 8 of the 9 evaluated parameters. This allows us to conclude that authors are currently reporting all these aspects, improving the quality of the studies and facilitating the transfer of basic research to clinical practice.

The results obtained and the outcomes highlighted in the application of mesenchymal stem cell-conditioned medium for bone regeneration in this review, allow us to indicate that in general, a positive and favorable effect on bone tissue regeneration with this intervention in human and animal models was observed. In animal models, the meta-analysis established an overall favorable effect of intervention with MSC-CM, indicating statistically significant differences in the percentage of bone regeneration between the MSC-CM groups and groups with other treatments. This demonstrates that the mechanism through which the MSC-CMs exert their biological effect, is primarily mediated by the action of growth factors, cytokines, and other constituent molecules, which stimulate and induce the migration of endogenous mesenchymal stem cells, endothelial cells and osteoprogenitor cells, promote their differentiation and expression of osteogenic and angiogenic markers, and stimulate angiogenesis, osteogenesis, repair and regeneration of bone tissue (Inukai et al., 2013; Katagiri et al., 2013; Katagiri et al., 2015; Katagiri et al., 2017b; Katagiri et al., 2017c; Kawai et al., 2015; Ogata et al., 2015; Osugi et al., 2012; Qin et al., 2016; Sanchooli et al., 2017). These effects obtained by the conditioned media are consistent with what has been previously reported in other studies where MSC-CM were applied for the regeneration of different tissues. Chen in 2008 and 2014 (Chen et al., 2008; Chen et al., 2014) mentioned that application of MSC-CM stimulated wound-healing due to the presence of high levels of cytokines that induced angiogenesis, migration and cell proliferation, thereby accelerating injury repair. Shen and Bangh in 2015 and 2014, respectively, indicated that factors present in MSC-CM have chemotactic properties, which are involved in the blood vessel formation and remodeling, angiogenesis stimulation and tissue repair (Shen et al., 2015; Bhang et al., 2014), Zhang et al., showed favorable results and effectiveness of MSC-CMs in repair and regeneration of cartilage (Zhang et al., 2016), Nakamura in 2015 showed that the secretome of mesenchymal stem cells accelerated regeneration of skeletal muscle (Nakamura et al., 2015), and Monsel and colleagues identified secretome effectiveness for the treatment of lung inflammatory diseases through activation of anti-inflammatory and antiapoptotic pathways (Monsel et al., 2016).

In most of the scientific papers evaluated in this review, the doses of MSC-CM used are expressed in volumetric units ranging from 10 μl to 6 ml, identifying a fairly wide range of dosage, which is likely to respond to variables such as the size of the bone defect and the scaffold used for the application of MSC-CM. But in our concept, to find a relationship between dose and therapeutic effectiveness, it is necessary to identify the concentration of total proteins contained in the applied conditioned medium, not just the volume used. Of the 21 articles included, 4 report the protein concentration (Linero & Chaparro, 2014; Tsuchiya et al., 2013; Wang et al., 2015; Xu et al., 2016); but only one makes a comparative analysis of bone regeneration of jaw defects where conditioned medium were applied with a protein concentration of 100 mg/ml vs twice protein concentration (200 mg/ml); identifying that there are no statistically significant differences in morphometric, radiographic and histological assessments (Linero & Chaparro, 2014). This finding suggests that the biological system has a saturation point where even if there are more proteins in the wound bed the therapeutic effect is not potentialized. However, we consider that more preclinical research is necessary to clarify the relationship between the dose, in terms of protein concentration, and the therapeutic effect.

The results found in studies performed in human models suggest a positive effect of MSC-CM application on bone regeneration (Katagiri et al., 2016; Katagiri et al., 2017a), blood vessel formation, osteogenesis, and bone tissue repair and regeneration without causing an inadequate inflammatory response or adverse effects. However, the evidence reported is not sufficient, and therefore, it is necessary to implement the development of phase I and II clinical trials to verify these effects in humans and allow for the implementation of MSC-CM for bone regeneration procedures in clinical practice.

With all this evidence, we can suggest that MSC-CM application will become a therapeutic alternative with a great potential for the treatment of bone defects. Implementing this new strategy will allow taking advantage of the clinical benefits of cell therapy, using a product free of cells that can be administered as a medicine, more easily adaptable to the therapeutic needs in individuals, allowing the translation of scientific research into clinical development, generating promising prospects for the thousands of patients who would benefit from this type of technological development.

Despite an exhaustive search of the literature, one of the main limitations of this review is the presence of bias related to the low number of published studies. In addition, 95% of the animal studies included were categorized with the high risk of bias due to the absence of randomization, concealment of sequence and blinding in the assessment of the results; therefore, it is likely to generate an overestimate of the treatment effect. It is important that the authors of preclinical studies begin to use the SYRCLE tool to improve the quality of their studies and reduce the biases that frequently occur in this type of research.

## Conclusion

The results of this systematic review indicate that the application of MSC-CM in the animal models is an effective therapy to stimulate bone regeneration and reduce healing time, thus favoring the quantity and quality of newly formed tissue without causing inflammatory reactions or adverse effects. The studies reported in the human models also suggest that MSC-CM improve the process of bone regeneration and may prove to be a safe and effective therapy. Thus, phase I and phase II clinical trials are required to support these findings and to support the application of conditioned medium as a potential therapeutic strategy for the treatment of bone defects.

## Data Availability

All data generated or analyzed during this study are included in this published article and supplementary information files.

## Appendix 1

### Electronic search strategy

1. conditioned medium.mp. (15296)
2. conditioned media.mp. (7810)
3. exp. Mesenchymal stromal cells/ (27495)
4. (Mesenchymal stromal cell* or marrow stromal cell*).mp. (33087)
5. (Mesenchymal stem cell* or marrow stem cell*).mp. (30926)
6. exp. Paracrine communication/(3591)
7. exp. Tissue engineering/ (28357)
8. exp. Exosomes/ (3266)
9. Exosome*.ti,ab. (4669)
10. exp. Regenerative medicine/ (4819)
11. Paracrine factor*.ab,ti. (1951)
12. Growth factor*.ab,ti. (294773)
13. cytokine.ab,ti. (161799)
14. exp. “Bone and Bones” (556202)
15. “bone*”.ab,ti. (568453)
16. exp. Bone Regeneration/ (21151)
17. bone repair.ab,ti. (2749)
18. bone defect.ab,ti. (3831)
19. bone healing.ab,ti. (5852)
20. “bone remodeling”.ab,ti. (7317)
21. bone consolidation.ab,ti. (227)
22. osteogenesis.ab,ti. (14955)
23. calvarial defect model.ab,ti. (200)
24. Osseointegration.ab,ti.(4731)
25. periodontal regeneration.ab,ti. (994)
26. Fractures.ab,ti. (116270)
27. (case report* or clinical study or clinical trial, all or clinical trial, phase i or clinical trial, phase ii or clinical trial, phase iii or clinical trial, phase iv or clinical trial or controlled clinical trial or multicenter study or pragmatic clinical trial or randomized controlled trial or experimental study or experiment* or in vivo or animal experiment or mouse model or rat or rabbit or dog or bovine).mp. (6108160)
28. 1 or 2 or 6 or 7 or 8 or 9 or 10 or 11 or 12 or 13 (491089)
29. 3 or 4 or 5 (42508)
30. 14 or 15 or 16 or 17 or 18 or 19 or 20 or 21 or 22 or 23 or 24 or 25 or 26 (1001553)
31. 27 and 28 and 29 and 30 (4158)
32. limit 31 to yr = “2000 -Current” (3994)
33. limit 32 to abstracts (3991)

**Embase (February 2018).**

#28 AND [embase]/lim NOT ([embase]/lim AND [medline]/lim) AND (‘animal experiment’/de OR ‘animal model’/de OR ‘controlled study’/de OR ‘experimental model’/de OR ‘human’/de OR ‘in vitro study’/de OR ‘in vivo study’/de) AND (2002,py OR 2003:py OR 2004:py OR 2005:py OR 2006:py OR 2007:py OR 2008:py OR 2009:py OR 2010:py OR 2011:py OR 2012:py OR 2013:py OR 2014:py OR 2015:py OR 2016:py OR 2017:py OR 2018:py) AND (‘article’/it OR ‘article in press’/it) (2169).

#27 #26 AND [embase]/lim NOT ([embase]/lim AND [medline]/lim) (5772).

#26 #24 AND #25 (13,931).

#25 #14 OR #15 OR #16 OR #17 OR #18 OR #19 OR

#20 OR #21 (816,966).

#24 #22 AND #23 (24,774).

#23 #2 OR #10 (66,923).

#22 #1 OR #3 OR #4 OR #5 OR #6 OR #7 OR #8 OR

#9 OR #11 OR #12 OR #13 (771,387).

#21 ‘bone tissue engineering’/exp. (72).

#20 ‘bone tissue engineering’:ab,ti (4717).

#19 ‘bone regeneration’:ab,ti (9413).

#18 ‘bone regeneration’ (25,557).

#17 ‘bone regeneration’/exp. (23,192).

#16 ‘bone remodeling’/exp. (25,599).

#15 ‘bone defect’/exp. (10,517).

#14 bone:ab,ti OR ‘bone repair’:ab,ti OR ‘bone healing’:ab,ti OR osteogenesis:ab,ti OR ‘calvarial defect model’:ab,ti OR osseointegration.ab,ti OR ‘periodontal regeneration’:ab,ti (807,277).

#13 ‘regenerative medicine’/exp. (11,407).

#12 ‘regenerative medicine’:ab,ti (15,277).

#11 ‘paracrine factor*’:ab,ti (2832).

#10 ‘stem cells mesenchymal’:ab,ti (210).

#9 ‘conditioned medium from cultures’ (67).

#8 ‘tissue engineering’:ab,ti (35,504).

#7 ‘cytokine production’ (130,076).

#6 ‘secretome’ (3297).

#5 ‘growth factor*’ (566,193).

#4 ‘growth factor*’:ab,ti (386,364).

#3 exosome*:ab,ti (10,178).

#2 ‘mesenchymal stem cell’ OR ‘mesenchymal stromal cell*’ OR ‘marrow stromal cell*’ OR ‘marrow stem cell’ (66,877).

#1 ‘conditioned medium’/exp. OR ‘conditioned medium’ OR ‘conditioned media’ (31,571).

**CENTRAL (OVID) EBM Reviews - Cochrane Central Register of Controlled Trials November 2017 (only human)**

1. exp. Culture media, conditioned/ (27)
2. Culture media, conditioned.ab,ti. (0)
3. exp. Mesenchymal stromal cells/ (77)
4. Mesenchymal stromal cells.ab,ti. (87)
5. exp. Paracrine communication/ (3)
6. Paracrine communication.ab,ti. (0)
7. exp. Intracellular signaling peptides/ and protein/ (14)
8. exp. Intracellular signaling peptides/ (1706)
9. “Intracellular signaling peptides and proteins”.ab,ti. (0)
10. “Intracellular Signaling Peptides and Proteins”/ (89)
11. exp. Tissue engineering/ (45)
12. exp. Exosomes/ (3)
13. Exosome*.ti,ab. (36)
14. exp. Regenerative medicine/ (7)
15. Regenerative medicine.ab,ti. (48)
16. Paracrine factor*.ab,ti. (20)
17. Growth factor*.ab,ti. (8259)
18. “Conditioned medium from cultures”.mp. (0)
19. 1 or 2 or 3 or 4 or 5 or 6 or 7 or 8 or 9 or 10 or 11 or 12 or 13 or 14 or 15 or 16 or 17 or 18 (10115)
20. exp. “Bone and Bones”/ (11456)
21. “bone and bones”.ab,ti. (0)
22. exp. Bone Regeneration/ (779)
23. “Alveolar bone loss”.ab,ti (42)
24. “bone development”.ab,ti. (31)
25. “bone lengthening”.ab,ti. (5)
26. “bone remodeling”.ab,ti. (378)
27. Fractures.ab,ti. (7530)
28. 20 or 21 or 22 or 23 or 24 or 25 or 26 or 27 (18546)
29. 19 and 28 (211)
30. limit 29 to (male and female and humans and (case reports or clinical study or clinical trial, all or clinical trial, phase i or clinical trial, phase ii or clinical trial, phase iii or clinical trial, phase iv or clinical trial or controlled clinical trial or multicenter study or pragmatic clinical trial or randomized controlled trial)) (193)

**SCOPUS (November 2017) (only human).**

(TITLE-ABS-KEY (“Culture media conditioned”) OR TITLE-ABS-KEY (“Mesenchymal stromal cells”) OR TITLE-ABS-KEY (“Paracrine communication”) OR TITLE-ABS-KEY (“Intracellular signaling peptides”) OR TITLE-ABS-KEY (“Tissue engineering”) OR TITLE-ABS-KEY (exosome*) OR TITLE-ABS-KEY (“Regenerative medicine”) OR TITLE-ABS-KEY (“Paracrine factor*”) OR TITLE-ABS-KEY (“Growth factor*”)) AND (TITLE-ABS-KEY (bone) OR TITLE-ABS-KEY (“Bone Regeneration”) OR TITLE-ABS-KEY (“Alveolar bone loss”) OR TITLE-ABS-KEY (“bone development”) OR TITLE-ABS-KEY (“bone lengthening”) OR TITLE-ABS-KEY (“bone remodeling”) OR TITLE-ABS-KEY (fracture)) AND (TITLE-ABS-KEY (human) OR TITLE-ABS-KEY (women) TITLE-ABS-KEY (men)) AND (TITLE-ABS-KEY (“clinical trial”) OR TITLE-ABS-KEY (“controlled clinical trial”) OR TITLE-ABS-KEY (“controlled study”) OR TITLE-ABS-KEY (“randomized controlled trial”) OR TITLE-ABS-KEY (“Cluster Analysis”) OR TITLE-ABS-KEY (“case report”)) AND (LIMIT-TO (SRCTYPE, “j “)) AND (LIMIT-TO (DOCTYPE, “ar “)) AND (LIMIT-TO (LANGUAGE, “English “) OR LIMIT-TO (LANGUAGE, “Spanish “)) AND (LIMIT-TO (PUBYEAR, 2017) OR LIMIT-TO (PUBYEAR, 2016) OR LIMIT-TO (PUBYEAR, 2015) OR LIMIT-TO (PUBYEAR, 2014) OR LIMIT-TO (PUBYEAR, 2013) OR LIMIT-TO (PUBYEAR, 2012) OR LIMIT-TO (PUBYEAR, 2011) OR LIMIT-TO (PUBYEAR, 2010) OR LIMIT-TO (PUBYEAR, 2009) OR LIMIT-TO (PUBYEAR, 2008) OR LIMIT-TO (PUBYEAR, 2007) OR LIMIT-TO (PUBYEAR, 2006) OR LIMIT-TO (PUBYEAR, 2005) OR LIMIT-TO (PUBYEAR, 2004) OR LIMIT-TO (PUBYEAR, 2003) OR LIMIT-TO (PUBYEAR, 2002) OR LIMIT-TO (PUBYEAR, 2001)) (533)

**Virtual health library (IBECS / LILACS / CUMED) (only human).**

tw:(tw:(((tw:(culture media conditioned)) OR (tw:(mesenchymal stromal cells)) OR (tw:(paracrine communication)) OR (tw:(intracellular signaling peptides)) OR (tw:(tissue engineering)) OR (tw:(regenerative medicine)) OR (tw:(growth factor)) OR (tw:(paracrine factor)) AND (tw:(bone)) OR (tw:(bone regeneration)) OR (tw:(alveolar bone loss)) OR (tw:(bone development)) OR (tw:(bone lengthening)) OR (tw:(bone remodeling)) OR (tw:(fracture)))) AND (instance:"regional”) AND (db:(“IBECS” OR “LILACS” OR “CUMED”) AND limit:(“humans” OR “female” OR “male” OR “adult”))) AND (instance:"regional”) (52).

**CENTRAL (OVID) EBM Reviews - Cochrane Central Register of Controlled Trials November 2017 (only animal)**

1. exp. Culture media, conditioned/ (27)
2. Culture media, conditioned.ab,ti. (0)
3. exp. Mesenchymal stromal cells/ (77)
4. Mesenchymal stromal cells.ab,ti. (87)
5. exp. Paracrine communication/ (3)
6. Paracrine communication.ab,ti. (0)
7. exp. Intracellular signaling peptides/ and protein/ (14)
8. exp. Intracellular signaling peptides/ (1706)
9. “Intracellular signaling peptides and proteins”.ab,ti. (0)
10. “Intracellular Signaling Peptides and Proteins”/ (89)
11. exp. Tissue engineering/ (45)
12. exp. Exosomes/ (3)
13. Exosome*.ti,ab. (36)
14. exp. Regenerative medicine/ (7)
15. Regenerative medicine.ab,ti. (48)
16. Paracrine factor*.ab,ti. (20)
17. Growth factor*.ab,ti. (8259)
18. “Conditioned medium from cultures”.mp. (0)
19. 1 or 2 or 3 or 4 or 5 or 6 or 7 or 8 or 9 or 10 or 11 or 12 or 13 or 14 or 15 or 16 or 17 or 18 (10115)
20. exp. “Bone and Bones”/ (11456)
21. “bone and bones”.ab,ti. (0)
22. exp. Bone Regeneration/ (779)
23. “Alveolar bone loss”.ab,ti. (42)
24. “bone development”.ab,ti. (31)
25. “bone lengthening”.ab,ti. (5)
26. “bone remodeling”.ab,ti. (378)
27. Fractures.ab,ti. (7530)
28. 20 or 21 or 22 or 23 or 24 or 25 or 26 or 27 (18546)
29. 19 and 28 (211)
30. limit 29 to (animals and yr = “2000 -Current” and animals) (178)

**SCOPUS (November 2017) (only animal).**

(TITLE-ABS-KEY (“Culture media conditioned”) OR TITLE-ABS-KEY (“Mesenchymal stromal cells”) OR TITLE-ABS-KEY (“Paracrine communication”) OR TITLE-ABS-KEY (“Intracellular signaling peptides”) OR TITLE-ABS-KEY (“Tissue engineering”) OR TITLE-ABS-KEY (exosome*) OR TITLE-ABS-KEY (“Regenerative medicine”) OR TITLE-ABS-KEY (“Paracrine factor*”) OR TITLE-ABS-KEY (“Growth factor*”)) AND (TITLE-ABS-KEY (bone) OR TITLE-ABS-KEY (“Bone Regeneration”) OR TITLE-ABS-KEY (“Alveolar bone loss”) OR TITLE-ABS-KEY (“bone development”) OR TITLE-ABS-KEY (“bone lengthening”) OR TITLE-ABS-KEY (“bone remodeling”) OR TITLE-ABS-KEY (fracture)) AND (TITLE-ABS-KEY (animal)) AND (TITLE-ABS-KEY (“animal experimental”)) AND (LIMIT-TO (SRCTYPE, “j ”)) AND (LIMIT-TO (DOCTYPE, “ar “)) AND (LIMIT-TO (PUBYEAR, 2017) OR LIMIT-TO (PUBYEAR, 2016) OR LIMIT-TO (PUBYEAR, 2015) OR LIMIT-TO (PUBYEAR, 2014) OR LIMIT-TO (PUBYEAR, 2013) OR LIMIT-TO (PUBYEAR, 2012) OR LIMIT-TO (PUBYEAR, 2011) OR LIMIT-TO (PUBYEAR, 2010) OR LIMIT-TO (PUBYEAR, 2009) OR LIMIT-TO (PUBYEAR, 2008) OR LIMIT-TO (PUBYEAR, 2007) OR LIMIT-TO (PUBYEAR, 2006) OR LIMIT-TO (PUBYEAR, 2005) OR LIMIT-TO (PUBYEAR, 2004) OR LIMIT-TO (PUBYEAR, 2003) OR LIMIT-TO (PUBYEAR, 2002) OR LIMIT-TO (PUBYEAR, 2001)) AND (LIMIT-TO (LANGUAGE, “English ”) OR LIMIT-TO (LANGUAGE, “Spanish ”)) (23)

**Virtual health library (IBECS / LILACS / CUMED) (only animal).**

tw:(((tw:(culture media conditioned)) OR (tw:(mesenchymal stromal cells)) OR (tw:(paracrine communication)) OR (tw:(intracellular signaling peptides)) OR (tw:(tissue engineering)) OR (tw:(regenerative medicine)) OR (tw:(growth factor)) OR (tw:(paracrine factor)) AND (tw:(bone)) OR (tw:(bone regeneration)) OR (tw:(alveolar bone loss)) OR (tw:(bone development)) OR (tw:(bone lengthening)) OR (tw:(bone remodeling)) OR (tw:(fracture)))) AND (instance:“regional”) AND (db:(“LILACS” OR “IBECS” OR “BBO” OR “SES-SP”) AND limit:(“animals”)) (30).

## Appendix 2

### Exclusion criteria

- Studies involving the application of MSC-CM for the regeneration of tissues other than bone.
- Studies using conditioned medium from cells other than mesenchymal stem cells.
- Studies that do not evaluate the amount of bone regeneration clinically, histologically and/or radiographically.

**Articles excluded after full-text review.**

**Table:**

Author	Title	Exclusion reason
Shang-Chun 2016 (97)	Exosomes from Human Synovial-Derived Mesenchymal Stem Cells Prevent Glucocorticoid-Induced Osteonecrosis of the Femoral Head in the Rat	This study evaluates the application of MSC-CM to prevent glucocorticoid-induced osteonecrosis. Does not evaluate bone regeneration.
Otsuru S 2018 (80)	Extracellular vesicles released from mesenchymal stromal cells stimulate bone growth in osteogenesis imperfecta	This study evaluates the application of EVs for bone growth in osteogenesis imperfecta, and does not present radiographic or histological analysis. Does not evaluate bone regeneration.
Li Y 2018 (98)	Human adipose-derived mesenchymal stem cell-conditioned media suppresses inflammatory bone loss in a lipopolysaccharide-induced murine model	This study evaluates the inhibition of bone loss mediated by lipopolysaccharides with the application of MSC-CM. Does not evaluate bone regeneration.
Byeon YE 2010 (99)	Paracrine effect of canine allogenic umbilical cord blood-derived mesenchymal stromal cells mixed with beta-tricalcium phosphate on bone regeneration in ectopic implantations	This study evaluates bone formation in ectopic places. It does not apply it in bone defects, therefore it does not evaluate bone regeneration.
Sakaguchi K 2017 (100)	Periodontal tissue regeneration using the cytokine cocktail mimicking secretomes in the conditioned media from human mesenchymal stem cells	This study applies a cytokine cocktail that mimics MSC-CM. It does not use CM derived from MSCs.
Pethő A 2018 (101)	Exosomes in Extracellular Matrix Bone Biology	This study corresponds to a literature review article.

## Appendix 3

Please insert Table 3 here

**Table 3:** Data extraction form

General description			pg & fig/table
Title of the study			
Authors			
Year			
ID o DOI			
Objective			
Protocol and registration			
Population and study environment			pg & fig/table
Description of the Population or animal model (species)			
Environment (includes location and social context) (human study)			
Total population at the beginning of the study Number of clusters (if applicable - Number and type of people per cluster)			
Age			
Sex			
Race / ethnicity			
Severity of the disease / co-morbidities			
Measurements by subgroup			
Methods			pg & fig/table
Study design			
Analysis unit (individual, cluster / groups)			
Start / end Date			
Total duration of the study			
Exclusion criteria			
Recruitment method (human)			
Calculation of sample size			
Approval of the ethics committee			
Risk assessment of bias for animal and human studies (clinical trials)			pg & fig/table
Domain	Type of risk	Supports	
	Low - High - “Unclear”		
Random sequence generation (Animal or human)			
Sequence concealment (Animal or human)			
Blinding of participants and staff (human)			
Blinding of outcome assessor (Animal or human)			
Incomplete outcome data (Animal or human)			
Selective outcome reporting (Animal or human)			
Baseline characteristics between groups (Animal or human)			
Random Housing (Animal)			
Random outcome assessment (animal)			
Blinding of the caregiver or researcher (Animal)			
**Intervention characteristics**			
Definition			
Administration method			
Intervention duration			
**Comparator group characteristics**			
Definition			
Administration method			
Intervention duration			
**Outcome**			pg & fig/table
Outcome name			
Definition			
How the measurement was applied (interview, email, telephone ...)			
Losses (%)			
Imputation of lost data			
Results (most important)%, mean, standard deviation.			
Statistical method used			
**Other information**			pg & fig/table
Conclusions			
Financing sources			
Note:			

## Appendix 4

Please insert Table 4 here.

**Table 4:** PRISMA checklist

Section/topic	#	Checklist item	Reported on page #	
**TITLE**				
Title	1	Identify the report as a systematic review, meta-analysis, or both.	Yes	
**ABSTRACT**				
Structured summary	2	Provide a structured summary including, as applicable: background; objectives; data sources; study eligibility criteria, participants, and interventions; study appraisal and synthesis methods; results; limitations; conclusions and implications of key findings; systematic review registration number.	Yes	
**INTRODUCTION**				
Rationale	3	Describe the rationale for the review in the context of what is already known.	Yes	
Objectives	4	Provide an explicit statement of questions being addressed with reference to participants, interventions, comparisons, outcomes, and study design (PICOS).	Yes	
**METHODS**				
Protocol and registration	5	Indicate if a review protocol exists, if and where it can be accessed (e.g., Web address), and, if available, provide registration information including registration number.	No	
Eligibility criteria	6	Specify study characteristics (e.g., PICOS, length of follow-up) and report characteristics (e.g., years considered, language, publication status) used as criteria for eligibility, giving rationale.	Yes	
Information sources	7	Describe all information sources (e.g., databases with dates of coverage, contact with study authors to identify additional studies) in the search and date last searched.	Yes	
Search	8	Present full electronic search strategy for at least one database, including any limits used, such that it could be repeated.	Yes	
Study selection	9	State the process for selecting studies (i.e., screening, eligibility, included in systematic review, and, if applicable, included in the meta-analysis).	Yes	
Data collection process	10	Describe method of data extraction from reports (e.g., piloted forms, independently, in duplicate) and any processes for obtaining and confirming data from investigators.	Yes	
Data items	11	List and define all variables for which data were sought (e.g., PICOS, funding sources) and any assumptions and simplifications made.	Yes	
Risk of bias in individual studies	12	Describe methods used for assessing risk of bias of individual studies (including specification of whether this was done at the study or outcome level), and how this information is to be used in any data synthesis.	Yes	
Summary measures	13	State the principal summary measures (e.g., risk ratio, difference in means).	Yes	
Synthesis of results	14	Describe the methods of handling data and combining results of studies, if done, including measures of consistency (e.g., I^2^) for each meta-analysis.	Yes	
**Section/topic**		**#**	**Checklist item**	**Reported on page #**
Risk of bias across studies		15	Specify any assessment of risk of bias that may affect the cumulative evidence (e.g., publication bias, selective reporting within studies).	yes
Additional analyses		16	Describe methods of additional analyses (e.g., sensitivity or subgroup analyses, meta-regression), if done, indicating which were pre-specified.	No
		**RESULTS**		
Study selection		17	Give numbers of studies screened, assessed for eligibility, and included in the review, with reasons for exclusions at each stage, ideally with a flow diagram.	Yes
Study characteristics		18	For each study, present characteristics for which data were extracted (e.g., study size, PICOS, follow-up period) and provide the citations.	Yes
Risk of bias within studies		19	Present data on risk of bias of each study and, if available, any outcome level assessment (see item 12).	Yes
Results of individual studies		20	For all outcomes considered (benefits or harms), present, for each study: (a) simple summary data for each intervention group (b) effect estimates and confidence intervals, ideally with a forest plot.	Yes
Synthesis of results		21	Present results of each meta-analysis done, including confidence intervals and measures of consistency.	Yes
Risk of bias across studies		22	Present results of any assessment of risk of bias across studies (see Item 15).	Yes
Additional analysis		23	Give results of additional analyses, if done (e.g., sensitivity or subgroup analyses, meta-regression [see Item 16]).	No
		**DISCUSSION**		
Summary of evidence		24	Summarize the main findings including the strength of evidence for each main outcome; consider their relevance to key groups (e.g., healthcare providers, users, and policy makers).	Yes
Limitations		25	Discuss limitations at study and outcome level (e.g., risk of bias), and at review-level (e.g., incomplete retrieval of identified research, reporting bias).	Yes
Conclusions		26	Provide a general interpretation of the results in the context of other evidence, and implications for future research.	Yes
		**FUNDING**		
Funding		27	Describe sources of funding for the systematic review and other support (e.g., supply of data); role of funders for the systematic review.	Yes

## Abbreviations

MSCs: Mesenchymal stem cells
MSC-CM: Mesenchymal stem cell-conditioned medium
SYRCLE: SYstematic Review Centre for Laboratory animal Experimentation
SMD: Standardized mean difference
CI: Confidence intervals
SFE: Maxillary sinus floor elevation
GBR: Guided bone regeneration
SP: Socket preservation
hMSCS: Human mesenchymal stem cells
rMSCS: Rat mesenchymal stem cells
BM: Bone marrow
Ad: Adipose tissue
hUCMSCs: Mesenchymal stem cells derived from human umbilical cord
F-hMSCs: Human fetal mesenchymal stem cells
Evs: Extracellular vesicles
B- TCP: Beta-tricalcium phosphate
BRONJ: Bisphosphonate-related osteonecrosis of the jaw
PBS: Phosphate buffered saline
DMEM: Dulbecco’s Modified Eagle Medium
DO: Distraction osteogenesis
CT: Computerized tomography
VEGF: Vascular endothelial growth factor

## References

[CR1] Akyurekli C, Le Y, Richardson RB, Fergusson D, Tay J, Allan DS (2015). A systematic review of preclinical studies on the therapeutic potential of Mesenchymal stromal cell-derived microvesicles. Stem Cell Rev.

[CR2] Ando Y, Matsubara K, Ishikawa J, Fujio M, Shohara R, Hibi H (2014). Stem cell-conditioned medium accelerates distraction osteogenesis through multiple regenerative mechanisms. Bone.

[CR3] Ankrum J, Karp JM (2010). Mesenchymal stem cell therapy: two steps forward, one step back. Trends Mol Med.

[CR4] Bari E (2019). Mesenchymal stem/stromal cell secretome for lung regeneration: the long way through “pharmaceuticalization” for the best formulation. J Control Release.

[CR5] Berthiaume F, Maguire T, Yarmush M (2011). Tissue engineering and regenerative medicine: history, Progress, and challenges. Annu Rev Chem Biomol Eng.

[CR6] Bertolai R, Catelani C, Aversa A, Rossi A, Giannini D, Bani D (2015). Bone graft and mesenchimal stem cells: clinical observations and histological analysis. Clin Cases Mineral Bone Metabolism.

[CR7] Bhang SH, Lee S, Shin JY, Lee TJ, Jang HK, Kim BS (2014). Efficacious and clinically relevant conditioned medium of human adipose-derived stem cells for therapeutic angiogenesis. Mol Ther.

[CR8] Byeon YE, Ryu HH, Park SS, Koyama Y, Kikuchi M, Kim WH (2010). Paracrine effect of canine allogenic umbilical cord blood-derived mesenchymal stromal cells mixed with beta-tricalcium phosphate on bone regeneration in ectopic implantations. Cytotherapy.

[CR9] Cancedda R, Giannoni P, Mastrogiacomo M (2007). A tissue engineering approach to bone repair in large animal models and in clinical practice. Biomaterials.

[CR10] Caplan AI, Correa D (2011). The MSC: an injury drugstore. Cell Stem Cell.

[CR11] Chang W, Kim R, Park SI, Jung YJ, Ham O, Lee J (2015). Enhanced Healing of Rat Calvarial Bone Defects with Hypoxic Conditioned Medium from Mesenchymal Stem Cells through Increased Endogenous Stem Cell Migration via Regulation of ICAM-1 TargetedmicroRNA-221. Mol Cells.

[CR12] Chaparro O, Linero I (2016). Regenerative Medicine: A New Paradigm in Bone Regeneration. Advanced Techniques in Bone Regeneration [Internet]. Intechopen.

[CR13] Chen L, Tredget EE, Wu PY, Wu Y (2008). Paracrine factors of mesenchymal stem cells recruit macrophages and endothelial lineage cells and enhance wound healing. PLoS One.

[CR14] Chen L, Xu Y, Zhao J, Zhang Z, Yang R, Xie J (2014). Conditioned medium from hypoxic bone marrow- derived Mesenchymal stem cells enhances wound healing in mice. PLoS One.

[CR15] Clough B, McCarley M, Krause U, Zeitouni S, Froese J, McNeill E (2015). Bone regeneration with Osteogenically enhanced Mesenchymal stem cells and their extracellular matrix proteins. J Bone Miner Res.

[CR16] D R (2010). Principles of bone grafting. Oral Maxillofacial Surg Clin N Am.

[CR17] de Santana T, Flores R, Bacha H, Gonçalves A, Mateus M, Rosa A (2015). Association of mesenchymal stem cells and osteoblasts for bone repair. Regen Med.

[CR18] Dimitriou R, Jones E, McGonagle D, Giannoudis PV (2011). Bone regeneration: current concepts and future directions. BMC Med.

[CR19] Fukuoka H, Suga H (2015). Hair regeneration treatment using adipose-derived stem cell conditioned medium: follow-up with Trichograms. Eplasty.

[CR20] Furuta T, Miyaki S, Ishitobi H, Ogura T, Kato Y, Kamei N (2016). Mesenchymal stem cell-derived Exosomes promote fracture healing in a mouse model. Stem Cells Transl Med.

[CR21] Gagnier JJ, Kienle G, Altman DG, Moher D, Sox H, Riley D (2013). CARE Group. The CARE guidelines: consensus-based clinical case reporting guideline development. BMJ Case Rep.

[CR22] Goulet JA, Senunas LE, DeSilva GL, Greenfield ML (1997). Autogenous iliac crest bone graft. Complications and functional assessment. Clin Orthop Relat Res.

[CR23] Higgins JPT, Green S (2011). Assessing risk of bias in included studies. Cochrane Handbook for Systematic Reviews of Interventions [Internet].

[CR24] Hirst JA, Howick J, Aronson JK, Roberts N, Perera R, Koshiaris C (2014). The need for randomization in animal trials: an overview of systematic reviews. PLoS One.

[CR25] Hooijmans CR, Rovers MM, de Vries RBM, Leenaars M, Ritskes-Hoitinga M, Langendam MW (2014). SYRCLE’s risk of bias tool for animal studies. BMC Med Res Methodol.

[CR26] Inoue T, Sugiyama M, Hattori H, Wakita H, Wakabayashi T, Ueda M (2013). Stem cells from human exfoliated deciduous tooth-derived conditioned medium enhance recovery of focal cerebral ischemia in rats. Tissue Eng Part A.

[CR27] Inukai T, Katagiri W, Yoshimi R, Osugi M, Kawai T, Hibi H (2013). Novel application of stem cell-derived factors for periodontal regeneration. Biochem Biophys Res Commun.

[CR28] Ishikawa J, Takahashi N, Matsumoto T, Yoshioka Y (2016). Factors secreted from dental pulp stem cells show multifaceted benefits for treating experimental rheumatoid arthritis. Bone.

[CR29] Ivanova G, Pereira T, Caseiro AR, Georgieva P, Maurício AC, Prasain DJ (2016). Metabolomic and Proteomic Analysis of the Mesenchymal Stem Cells’ Secretome. Metabolomics - Fundamentals and Applications. InTech.

[CR30] Izumoto-Akita T, Tsunekawa S, Yamamoto A, Uenishi E, Ishikawa K, Ogata H (2015). Secreted factors from dental pulp stem cells improve glucose intolerance in streptozotocin-induced diabetic mice by increasing pancreatic β-cell function. BMJ Open Diabetes Res Care.

[CR31] Pawitan Jeanne Adiwinata (2014). Prospect of Stem Cell Conditioned Medium in Regenerative Medicine. BioMed Research International.

[CR32] Katagiri W, Kawai T, Osugi M, Sugimura-Wakayama Y, Sakaguchi K, Kojima T (2017). Angiogenesis in newly regenerated bone by secretomes of human mesenchymal stem cells. Maxillofacial Plastic Reconstructive Surg.

[CR33] Katagiri W, Masashi O, Takamasa K, Ueda M (2013). Novel cell-free regeneration of bone using stem cell-derived growth factors. Int J Oral Maxillofac Implants.

[CR34] Katagiri W, Osugi M, Kawai T, Hibi H (2016). First-in-human study and clinical case reports of the alveolar bone regeneration with the secretome from human mesenchymal stem cells. Head Face Med.

[CR35] Katagiri W, Osugi M, Kinoshita K, Hibi H (2015). Conditioned Medium From Mesenchymal Stem Cells Enhances Early Bone Regeneration After Maxillary Sinus Floor Elevation in Rabbits. Implant Dent.

[CR36] Katagiri W, Sakaguchi K, Kawai T, Wakayama Y, Osugi M, Hibi H (2017). A defined mix of cytokines mimics conditioned medium from cultures of bone marrow-derived mesenchymal stem cells and elicits bone regeneration. Cell Prolif.

[CR37] Katagiri W, Watanabe J, Toyama N, Osugi M, Sakaguchi K, Hibi H (2017). Clinical study of bone regeneration by conditioned medium from Mesenchymal stem cells after maxillary sinus floor elevation. Implant Dent.

[CR38] Kawai T, Katagiri W, Osugi M, Sugimura Y, Hibi H, Ueda M (2015). Secretomes from bone marrow-derived mesenchymal stromal cells enhance periodontal tissue regeneration. Cytotherapy.

[CR39] Klyushnenkova E, Mosca JD, Zernetkina V, Majumdar MK, Beggs KJ, Simonetti DW (2005). T cell responses to allogeneic human mesenchymal stem cells: immunogenicity, tolerance, and suppression. J Biomed Sci.

[CR40] L PK (2019). The mesenchymal stem cell secretome: a new paradigm towrds cell-free therapeutic mode in regenerative medicine. Cytokine Growth Factor Rev.

[CR41] Le Blanc K, Davies LC (2018). MSCs-cells with many sides. Cytotherapy.

[CR42] Lee SM, Lee SC, Kim SJ (2014). Contribution of human adipose tissue-derived stem cells and the secretome to the skin allograft survival in mice. J Surg Res.

[CR43] Li Y, Gao X, Wang J (2018). Human adipose-derived mesenchymal stem cell-conditioned media suppresses inflammatory bone loss in a lipopolysaccharide-induced murine model. Exp Ther Med.

[CR44] Liang X, Ding Y, Zhang Y, Tse HF, Lian Q (2014). Paracrine mechanisms of Mesenchymal stem cell-based therapy: current status and perspectives. Cell Transplant.

[CR45] Linero I, Chaparro O (2014). Paracrine effect of Mesenchymal stem cells derived from human adipose tissue in bone regeneration. PLoS One.

[CR46] Ma H, Wu Y, Zhang W (2013). The effect of mesenchymal stromalcells on doxorubicin-induced nephropathy in rats. Cytotherapy.

[CR47] Mason C, Dunnill P (2008). A brief definition of regenerative medicine. Regen Med.

[CR48] Matsushita Y, Ishigami M, Matsubara K, Kondo M, Wakayama H, Goto H (2017). Multifaceted therapeutic benefits of factors derived from stem cells from human exfoliated deciduous teeth for acute liver failure in rats. J Tissue Eng Regen Med.

[CR49] Mita T, Furukawa-Hibi Y, Takeuchi H, Hattori H, Yamada K, Hibi H (2015). Conditioned medium from the stem cells of human dental pulp improves cognitive function in a mouse model of Alzheimer's disease. Behav Brain Res.

[CR50] Moher D, Liberati A, Tetzlaff J, Altman DG, PRISMA group (2009). Preferred Reporting Items for Systematic Reviews and Meta-Analyses: The PRISMA Statement. PLoS Med.

[CR51] Monaco E, Bionaz M, Hollister SJ, Wheeler MB (2011). Strategies for regeneration of the bone using porcine adult adipose-derived mesenchymal stem cells. Theriogenology.

[CR52] Monsel A, Zhu YG, Gudapati V, Lim H, Lee JW (2016). Mesenchymal stem cell derived Secretome and extracellular vesicles for acute lung injury and other inflammatory lung diseases. Expert Opin Biol Ther.

[CR53] Muhammad SA, Nordin N, Fakurazi S (2018). Regenerative potential of secretome from dental stem cells: a systematic review of preclinical studies. Rev Neurosci.

[CR54] Nakamura Y, Miyaki S, Ishitobi H, Matsuyama S, Nakasa T, Kamei N (2015). Mesenchymal-stem-cell-derived exosomes accelerate skeletal muscle regeneration. FEBS Lett.

[CR55] Ogata K, Katagiri W, Osugi M, Kawai T, Sugimura Y, Hibi H (2015). Evaluation of the therapeutic effects of conditioned media from mesenchymal stem cells in a rat bisphosphonate-related osteonecrosis of the jaw-like model. Bone.

[CR56] Oryan A, Alidadi S, Moshiri A (2013). Current concerns regarding healing of bone defects. Hard Tissue.

[CR57] Osugi M, Katagiri W, Yoshimi Y, Inukai T, Hibi H, Ueda M (2012). Conditioned media from Mesenchymal stem cells enhanced bone regeneration in rat Calvarial bone defects. Tissue Eng Part A.

[CR58] Otsuru S, Desbourdes L, Guess AJ, Hofmann TJ, Relation T, Kaito T (2018). Extracellular vesicles released from mesenchymal stromal cells stimulate bone growth in osteogenesis imperfecta. Cytotherapy.

[CR59] Padial M, O’Valle F, Lanis A, Mesa F, Dohan D, LayWang H (2015). Clinical application of Mesenchymal stem cells and novel supportive therapies for Oral bone regeneration. Biomed Res Int.

[CR60] Pethő A, Chen Y, George A (2018). Exosomes in extracellular matrix bone biology. Curr Osteoporos Rep.

[CR61] Pilipchuk SP, Plonka AB, Monje A, Taut AD, Lanis A, Kang B (2015). Tissue engineering for bone regeneration and osseointegration in the oral cavity. Dent Mater.

[CR62] Porzionato A (2019). Intratracheal administration of clinical-grade mesenchymal stem cell-derived extracellular veesicles reduces lung injury in a rat model of bronchopulmonary dysplasia. Am J Physiol Lung Cell Mol Physiol.

[CR63] Qin Y, Wang L, Gao Z, Chen G, Zhang C (2016). Bone marrow stromal/stem cellderived extracellular vesicles regulate osteoblast activity and differentiation in vitro and promote bone regeneration in vivo. Sci Rep.

[CR64] Ramamoorthi M, Bakkar M, Jordan J, Tran S (2015). Osteogenic potential of dental Mesenchymal stem cells in preclinical studies: a systematic review using modified ARRIVE and CONSORT guidelines. Stem Cells Int.

[CR65] Review Manager (RevMan) [Computer program] (2014). Version 5.3. Copenhagen: The Nordic Cochrane Centre, The Cochrane Collaboration.

[CR66] Rosset P, Deschaseaux F, Layrolle P (2014). Cell therapy for bone repair. Orthop Traumatol Surg Res.

[CR67] Saeed H, Ahsan M, Saleem Z, Iqtedar M, Islam M, Danish Z (2016). Mesenchymal stem cells (MSCs) as skeletal therapeutics–an update. J Biomed Sci.

[CR68] Sakaguchi K, Katagiri W, Osugi M, Kawai T, Sugimura-Wakayama Y, Hibi H (2017). Periodontal tissue regeneration using the cytokine cocktail mimicking secretomes in the conditioned media from human mesenchymal stem cells. Biochem Biophys Res Commun.

[CR69] Sanchooli T, Norouzian M, Ardeshirylajimi A, Ghoreishi SK, Abdollahifar MA, Nazarian H (2017). Adipose derived stem cells conditioned Media in Combination with bioceramic-collagen scaffolds improved Calvarial bone healing in hypothyroid rats. Iran Red Crescent Med J.

[CR70] Shang-Chun G, Shi-Cong T, Wen-Jing Y, Xin Q, Jia-Gen S, Chang-Qing Z (2016). Exosomes from human synovial-derived Mesenchymal stem cells prevent glucocorticoid-induced osteonecrosis of the femoral head in the rat. Int J Biol Sci.

[CR71] Shen C, Lie P, Miao T, Yu M, Lu Q, Feng T (2015). Conditioned medium from umbilical cord mesenchymal stem cells induces migration and angiogenesis. Mol Med Rep.

[CR72] Shimojima C, Takeuchi H, Jin S, Parajuli B, Hattori H, Suzumura A (2016). Conditioned medium from the stem cells of human exfoliated deciduous teeth ameliorates experimental autoimmune encephalomyelitis. J Immunol.

[CR73] Shrivats A, Alvarez P, Schutte L, Hollinger J. Bone Regeneration. In: Lanza R VJ, Langer R, editor. Principles of Tissue Engineering. 4th ed: Elsevier; 2014. p. 1201–21.

[CR74] Spees JL, Lee RH (2016). Gregory CA mechanisms of mesenchymal stem/stromal cell function. Stem Cell Res Ther.

[CR75] Tatullo M, Marrelli M, Paduano F (2015). The regenerative medicine in Oral and maxillofacial surgery: the Most important innovations in the clinical application of Mesenchymal stem cells. Int J Med Sci.

[CR76] Tran C, Damaseer MS (2015). Stem cells as drug delivery methods: application of stem cell secretome for regeneration. Adv Drug Deliv Rev.

[CR77] Tsuchiya S, Hara K, Ikeno M, Okamoto Y, Hibi H, Ueda M (2013). Rat bone marrow stromal cell- conditioned medium promotes early osseointegration of titanium implants. Int J Oral Maxillofac Implants.

[CR78] Tsuchiya S, Ohmori M, Hara K, Fujio M, Ikeno M, Hibi H (2015). An experimental study on guided bone regeneration using a polylactide-co-glycolide membrane- immovilized conditioned medium. Int J Oral Maxillofac Implants.

[CR79] Wakayama H, Hashimoto N, Matsushita Y, Matsubara K, Yamamoto N, Hasegawa Y (2015). Factors secreted from dental pulp stem cells show multifaceted benefits for treating acute lung injury in mice. Cytotherapy.

[CR80] Wang C-Y, Yang H-B, Hsu H-S, Chen L-L, Tsai C-C, Tsai K-S (2012). Mesenchymal stem cell-conditioned medium facilitates angiogenesis and fracture healing in diabetic rats. J Tissue Eng Regen Med.

[CR81] Wang J, Ding F, Gu Y, Liu J, Gu X (2009). Bone marrow mesenchymal stem cells promote cell proliferation and neurotrophic function of Schwann cells in vitro and in vivo. Brain Res.

[CR82] Wang Kui-Xing, Xu Liang-Liang, Rui Yun-Feng, Huang Shuo, Lin Si-En, Xiong Jiang-Hui, Li Ying-Hui, Lee Wayne Yuk-Wai, Li Gang (2015). The Effects of Secretion Factors from Umbilical Cord Derived Mesenchymal Stem Cells on Osteogenic Differentiation of Mesenchymal Stem Cells. PLOS ONE.

[CR83] Wang Shihua, Qu Xuebin, Zhao Robert (2012). Clinical applications of mesenchymal stem cells. Journal of Hematology & Oncology.

[CR84] Wen Y, Jiang B, Cui J, Li G, Yu M, Wang F (2013). Superior osteogenic capacity of different mesenchymal stem cells for bone tissue engineering. Oral Surg Oral Med Oral Pathol Oral Radiol.

[CR85] Xu J, Wang B, Sun Y, Wu T, Liu Y, Zhang J (2016). Human fetal mesenchymal stem cell secretome enhances bone consolidation in distraction osteogenesis. Stem Cell Res Ther.

[CR86] Yamaguchi S, Shibata R, Yamamoto N, Nishikawa M, Hibi H, Tanigawa T, et al. Dental pulp-derived stem cell conditioned medium reduces cardiac injury following ischemia-reperfusion. Sci Rep. 2015;5(16295). 10.1038/srep16295. 10.1038/srep16295 PMC4635346 26542315

[CR87] Yang X, Zhu TY, Wen LC, Cao YP, Liu C, Cui YP (2015). Intraarticular Injection of Allogenic Mesenchymal Stem Cells has a Protective Role for the Osteoarthritis. Chin Med J (Engl).

[CR88] Yoshikawa T, Mitsuno H, Nonaka I, Sen Y, Kawanishi K, Inada Y (2008). Wound therapy by marrow mesenchymal cell transplantation. Plast Reconstr Surg.

[CR89] Zhang S, Chu WC, Lai RZ, Lim SK, Hui JH, Toh WS (2016). Exosomes derived from human embryonic mesenchymal stem cells promote osteochondral regeneration. Osteoarthr Cartil.

[CR90] Zhang W, Liu XC, Yang L, Zhu DL, Zhang YD, Chen Y (2013). Wharton's jelly-derived mesenchymal stem cells promote myocardial regeneration and cardiac repair after miniswine acute myocardial infarction. Coron Artery Dis.

